# Asymmetric Mode of Ca^2+^-S100A4 Interaction with Nonmuscle Myosin IIA Generates Nanomolar Affinity Required for Filament Remodeling

**DOI:** 10.1016/j.str.2012.02.002

**Published:** 2012-04-04

**Authors:** Paul R. Elliott, Andrew F. Irvine, Hyun Suk Jung, Kaeko Tozawa, Martyna W. Pastok, Remigio Picone, Sandip K. Badyal, Jaswir Basran, Philip S. Rudland, Roger Barraclough, Lu-Yun Lian, Clive R. Bagshaw, Marina Kriajevska, Igor L. Barsukov

**Affiliations:** 1 Institute of Integrative Biology, BioSciences Building, Crown Street, University of Liverpool, Liverpool L69 7ZB, UK; 2 Department of Biochemistry, University of Leicester, Henry Wellcome Building, Lancaster Road, Leicester LE1 9HN, UK; 3 Department of Cancer Studies and Molecular Medicine, Robert Kilpatrick Clinical Sciences Building, University of Leicester, Leicester LE2 7LX, UK; 4 Division of Electron Microscopic Research, Korea Basic Science Institute, 52 Eoeun-dong, Daejeon 305-333, Korea; 5 MRC Laboratory for Molecular Cell Biology, University College London, London WC1E 6BT, UK; 6 Centre for Mathematics and Physics in the Life Sciences and Experimental Biology, University College London, London WC1E 6BT, UK; 7 London Centre for Nanotechnology, London WC1H 0AH, UK

## Abstract

Filament assembly of nonmuscle myosin IIA (NMIIA) is selectively regulated by the small Ca^2+^-binding protein, S100A4, which causes enhanced cell migration and metastasis in certain cancers. Our NMR structure shows that an S100A4 dimer binds to a single myosin heavy chain in an asymmetrical configuration. NMIIA in the complex forms a continuous helix that stretches across the surface of S100A4 and engages the Ca^2+^-dependent binding sites of each subunit in the dimer. Synergy between these sites leads to a very tight association (K_D_ ∼1 nM) that is unique in the S100 family. Single-residue mutations that remove this synergy weaken binding and ameliorate the effects of S100A4 on NMIIA filament assembly and cell spreading in A431 human epithelial carcinoma cells. We propose a model for NMIIA filament disassembly by S100A4 in which initial binding to the unstructured NMIIA tail initiates unzipping of the coiled coil and disruption of filament packing.

## Graphical abstract

**Graphical Abstract:**

**Figure:**
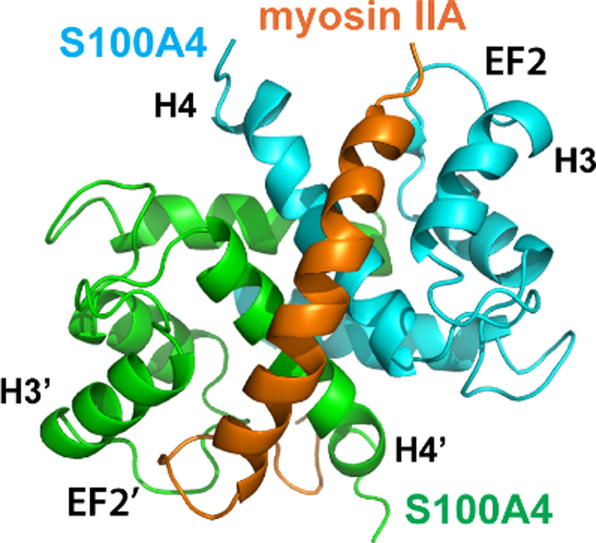

**Highlights:**

► S100A4 dimer binds single-myosin IIA molecule in an asymmetrical mode ► Synergy between Ca-dependent sites in S100A4 leads to high-affinity interaction ► Electron microscopy shows that two S100A4 dimers bind to the myosin coiled coil ► S100A4 has direct effect on formation of stress fibers and cell migration

## Introduction

Many fundamental processes in cells such as adhesion, migration, and proliferation are force dependent. Maintenance of cell polarity and cell-cell and cell-matrix contacts requires continuous tension. In migrating cells, adhesion complexes are established under the influence of tension and coordinate traction for cell propulsion. The intracellular force is generated by nonmuscle myosin II (NMII), which is ubiquitously expressed in eukaryotic cells (Vicente-Manzanares et al., 2009). NMII has two main isoforms, NMIIA and NMIIB, with distinctly different properties. Recent studies in several cell lines, such as CHO-K1, COS-7, and B16, showed that NMIIA is more dynamic and is involved in the initial adhesion stages at the leading edge of the cell, as well as the force-controlled disassembly of the adhesion complexes at the trailing edge. In contrast, NMIIB is mainly localized in the central zones and the cell rear, where it generates persistent tension required for the maintenance of stable adhesions (Vicente-Manzanares et al., 2007).

The differences in the localization of the NMII isoforms in CHO-K1, COS-7, and B16 cells are associated with the C-terminal coiled-coil region (Vicente-Manzanares et al., 2008). This region is required for the formation of the bipolar myosin filaments that bundle actin into stress fibers and generate sliding motion of the actin filaments. The last ∼200 residues of the coiled-coil region contain the assembly competent domain (ACD) that is involved in the regulation of the filament assembly by phosphorylation. An additional regulatory mechanism is associated with the small Ca^2+^-binding protein S100A4 (Garrett et al., 2006; Tarabykina et al., 2007) that selectively associates with ACD of NMIIA, but not NMIIB. In vitro, S100A4 binding causes myosin filaments and rod fragments to dissociate (Ford et al., 1997; Kriajevska et al., 2000; Li et al., 2003; Badyal et al., 2011). The level of S100A4 mRNA and protein is upregulated in a range of migrating cells and in metastases. The role of S100A4 as a metastasis promoter has been demonstrated in mouse and rat models (Davies et al., 1993; Ambartsumian et al., 1996; Davies et al., 1996). It is important to note that many cell types do not have a clear separation of NMIIA and NMIIB distribution and activities as the analyzed CHO-K1, COS-7, and B16 cell lines. This suggests that S100A4 may play a prominent role only in certain unmodified or transformed cell types.

S100A4 belongs to a large family of dimeric EF-hand calcium-binding proteins (Santamaria-Kisiel et al., 2006). Similar to other S100 proteins, Ca^2+^ binding induces a conformational switch that exposes a binding site in the cleft between helices H3 and H4 of the C-terminal EF hand in each of the subunits of the S100A4 dimer. Although the structures of the apo- and Ca^2+^-bound forms have been solved (Vallely et al., 2002; Gingras et al., 2008; Malashkevich et al., 2008), the structure of the S100A4/NMIIA complex remained elusive. In the absence of direct structural information, conclusions on the size and location of the binding site have been drawn from binding studies that involved a range of myosin fragments. Myosin peptides containing as few as 15 residues were reported to bind to S100A4 with micromolar affinity that is compatible with a site involving a single EF-hand cleft (Malashkevich et al., 2008). However, these results were influenced by the use of the fluorescent label that interacts with S100A4 and directly contributes to the binding (Badyal et al., 2011).

Here, using unmodified myosin fragments, we have identified the full binding region of S100A4 in myosin and have solved the structure of the complex. We demonstrate that both Ca^2+^-dependent sites of the S100A4 dimer contribute to the binding of a single NMIIA heavy chain resulting in an asymmetrical 1:2 NMIIA/S100A4 complex with nanomolar affinity. Using the structure of the complex, we designed mutations that interfere with the coupling between the binding sites, while leaving the EF2-hand clefts unmodified. The mutations dramatically reduce the affinity, demonstrating the dominant role of synergy between the two EF2-hand sites of the S100A4 dimer in the binding mechanism. Using the same S100A4 mutants, we demonstrate a direct effect of S100A4 on cell migration, cell spreading, and assembly of myosin filaments. Electron microscopy (EM) directly shows the association of a myosin coiled-coil fragment with two S100A4 dimers, in agreement with the 1:2 NMIIA:S100A4 stoichiometry.

## Results

### Mapping the S100A4-Binding Site on NM-MHC IIA

**Figure 1:**
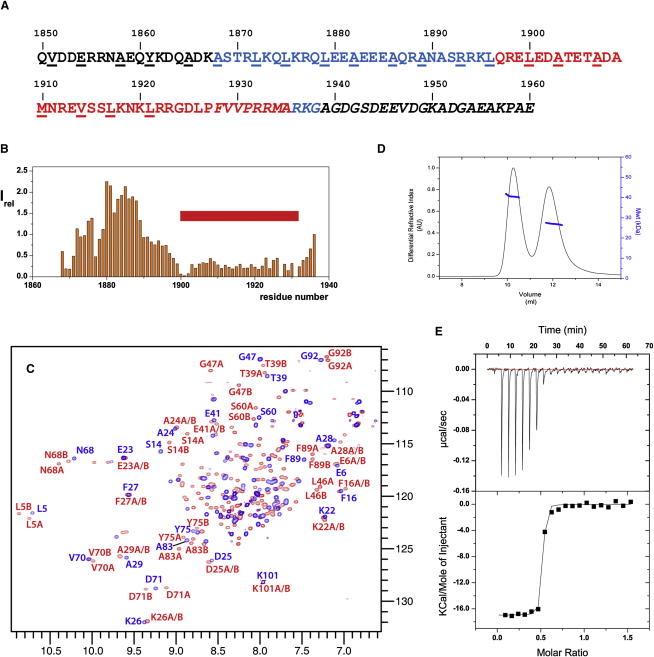
Interaction of S100A4 with NMIIA (A) Sequence of myosin fragments used in the binding site mapping. M111 corresponds to the whole sequence, M70 is highlighted in blue and red, and M39 in red. Residues in italic identify the unstructured C-terminal region of myosin. Residues in *a* and *d* positions of the heptad repeat that are critical for the coiled-coil stability are underlined. (B) Intensities of ^1^H,^15^N-HSQC cross-peaks of uniformly ^15^N-labeled M70 in complex with u-S100A4 normalized on the intensity for the last residue G1938. Low intensities correspond to the residue immobilized by direct contact with S100A4. M39 location is indicated by the red rectangle (C) ^1^H,^15^N-HSQC spectrum of ^15^N-labeled S100A4 in the free form (blue) and in the presence of 0.5 mole equivalent of u-M39 (red). Doubling of the cross-peak demonstrates asymmetric environment for equivalent residues of the S100A4 dimer. Colors of the labels indicate free (blue) and bound (red) forms. Residues are labeled A, B, or A/B to denote the monomer subunit that gives rise to the signal. (D) Gel filtration elution profile of the SEC-MALLS experiment for the 1:1 molar ratio used to determine the molecular weight of S100A4/M111 complex. Both the complex (peak I) and the free form of S100A4 (peak II) are observed. The molecular weight of 39.3 kDa measured for the M111/S100A4 peak is in a very good agreement with 40.1 kDa expected for the 1:2 complex. (E) ITC record of S100A4 titration with M39 at 10 μM S100A4 in the cell and 75 μM of M39 in the syringe. The solid line shows the fitting to the single site-binding model with K_D_ of 4 nM. See Figure S1 for further binding measurements. See also Table S1.

The extent of the S100A4-binding site in NMIIA has been controversial (Kriajevska et al., 1998; Malashkevich et al., 2008; Badyal et al., 2011). Recently, we used NMR to demonstrate that the unstructured region F1926–A1939 that immediately follows the coiled-coil region is essential for the interaction with S100A4 (Badyal et al., 2011). However, the large size of the myosin fragment M111 (residues Q1850–E1960) used in the analysis did not allow us to determine the N-terminal boundary of the binding site because signals of the residues from the coiled-coil region were not observed (Badyal et al., 2011). Based on these previous data, we reduced the size of the fragment by removing the residues not in contact with S100A4 (Figure 1A), generating an M70 fragment (residues A1868–G1938).

The ^1^H,^15^N-HSQC spectrum of M70 at elevated temperature of 40°C corresponds to a highly dynamic random coil state with uniform intensities for the majority of the cross-peaks (see Figure S1A available online). The backbone ^13^C chemical shift values support this conclusion (Figure S2G). The addition of S100A4 leads to a dramatic increase of the chemical shift dispersion and broadening of the cross-peaks in a continuous region E1899–R1933 (Figures 1B and S1A). This signifies direct contact of this region with S100A4 and identifies it as the binding site. Based on this mapping, we investigated a peptide, M39 (residues Q1897–A1935), that has two residue extensions at the N and C termini of the S100A4-interacting region. Both M70 and M39 induce changes in the ^1^H,^15^N-HSQC spectra of Ca^2+^-bound S100A4 (Figure S1B), confirming the similarity of the interactions for the two fragments.

Unexpectedly, for approximately half of the residues, two HSQC peaks with distinctly different chemical shifts are observed for the ^15^N-labeled S100A4 in the complex with unlabeled myosin, in contrast to one peak per residue detected for the free protein (Figure 1C). The doubled cross-peaks appear on the first addition of the peptide and remain at equal relative intensity throughout the S100A4 titration with M39, corresponding to a slow exchange regime due to the high affinity of the interaction. The signals of the free form disappear completely when the M39 monomer concentration is above the concentration of the S100A4 dimer, and no further changes of the S100A4 spectra are observed. These spectral changes and the concentration dependence suggest that a single M39 molecule binds to the S100A4 dimer, resulting in an asymmetrical 1:2 M39/S100A4 complex. In this arrangement, some sequentially identical sites of the two monomers in the S100A4 dimer would be in contact with different parts of the peptide, making them structurally nonequivalent and leading to different chemical shifts.

The similarity between the spectra of S100A4 complexes with M39 and M70 shows that asymmetric binding is a general characteristic of S100A4 interaction with myosin. For shorter myosin fragments, only a single peak was detected for each S100A4 residue in the complex, although a number of peaks were severely broadened even at high excess of myosin over S100A4 (Malashkevich et al., 2008; Badyal et al., 2011). Furthermore, chemical shifts of S100A4 changed gradually on the myosin peptide addition, demonstrating weaker interaction, as expected for the incomplete binding region. The residual broadening of the S100A4 resonances in the complex with short peptides is likely to be caused by the exchange of the peptide between equivalent asymmetrical arrangements on the S100A4 dimer.

Independent direct evidence for the 1:2 stoichiometry of S100A4 complexes with a myosin fragment was obtained from multi-angle laser light-scattering experiments (MALLS), coupled with refractive index and absorbance detection, that provide absolute molecular weights of proteins independently of their shape and folding state (Wyatt, 1993). The S100A4/myosin complex was resolved from the free protein using size-exclusion chromatography (SEC) in a SEC-MALLS setup, although no conclusion on the molecular weights was derived from the elution profiles due to their strong dependence on the shape of the proteins. For both S100A4/M111 (Figure 1D) and S100A4/M70 (Figure S2) complexes, uniform distribution of the molecular weight was observed across the peak of the complex. The measured molecular weights of 38.3 ± 0.11 kDa and 34.2 ± 0.15 kDa for complexes with M111 and M70, respectively, were close to the respective values of 40.1 and 34.3 predicted for the 1:2 S100A4/myosin stoichiometry of these fragments.

The affinity of the interaction was too high to determine the binding constant from the NMR titration, although it clearly showed 1:2 stoichiometry. A large enthalpy release on binding allowed us to record good quality isothermal titration calorimetry (ITC) data at protein concentrations as low as 10 μM, but even at these concentrations, the transition between the free and the bound state was sharp, and the binding isotherm had a small number of points in the transition region (Figure 1E). A single binding site model resulted in a good fit with a K_D_ of 5.9 ± 1.3 nM (n = 3) and M39/S100A4 ratio of 0.55 ± 0.06. The stoichiometry of the interaction is well defined and agrees with the NMR and MALLS measurement. The determined K_D_, however, is only an upper-limit value because the number of points in the transition area is too small for accurate fitting. At least 10-fold reduction in the protein concentration is required for the accurate measurement, which is not possible due to the limit of the instrument sensitivity.

To support the ITC measurements, we used the surface plasmon resonance (SPR) method that can be conducted at lower concentrations. The titration sensorgrams at nanomolar concentrations of M39 show a relatively fast association rate constant and a slow k_off_. Simultaneous fitting of the sensorgrams to a single site-binding model results in a K_D_ of 0.24 ± 0.07 nM (n = 3) (Figure S1H). The NMR, MALLS, and ITC results confirm earlier indications of the 1:2 stoichiometry for NMIIA/S100A4 complex deduced from the concentration of S100A4 required to solubilize a M200 (Q1761–E1960) filament (Badyal et al., 2011). In that work we also showed that an M32 peptide (A1907-G1938) bound to S100A4 with a K_D_ of 3 μM. Using similar kinetic assays (Badyal et al., 2011), we determine here (Figures S1I–S1L) that M39 binds to S100A4 with a K_D_ of ∼0.1 nM and a k_off_ of ≈0.001 s^−1^, in line with the SPR studies above. These data support the notion that the K_D_ determined by ITC is best viewed as an upper limit, and the true K_D_ is <1 nM. We, therefore, conclude the K_D_ is ≤1 nM as a limit compatible with all methods.

### Structure of M39/S100A4 Complex

To determine the structure of the S100A4/NMIIA complex, we used synthetic unlabeled M39, recombinant unlabeled and ^13^C,^15^N-labeled M70, and recombinant unlabeled and ^13^C,^15^N-labeled S100A4 to produce complexes where only one of the component is isotope labeled for selective detection of resonances from each of the components. The chemical shifts of S100A4 in both the M39 and M70 complexes, as well as the shifts of M39 and the corresponding region of M70 are close, demonstrating structural similarity. This agrees with the lack of direct contacts from the N-terminal (A1868–Q1897) part of M70 that are absent in M39 and allows us to combine the data for the two complexes when solving the structure.

Most of the resonances of the N-terminal pseudo-EF1 hand region 1–44 of the two subunits of S100A4 dimer in the complex are close and not resolved in 3D spectra, with an exception of a small number of resonances primarily of backbone HN groups. Additionally, the NOE patterns for the resolved resonances in this region are very similar, showing that the two subunits have similar structures in this region. In contrast, the resonances of the EF2-hand region 45–95 are distinctly different in the two subunits, indicating possible structural differences. To account for this, we applied symmetry restraints only for the N-terminal part. The resonances of the C-terminal region 96–101 are sharp and identical for both subunits, demonstrating that this part is unstructured and dynamic.

**Figure 2:**
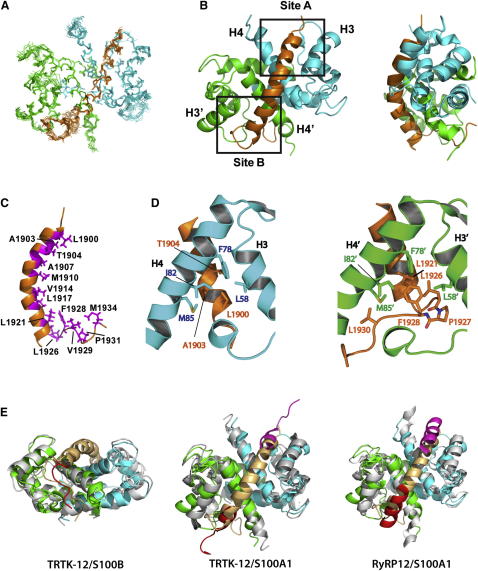
Structure of M39/S100A4 Complex (A) Superposition of the backbone atoms for ten lowest-energy structures of M39/S100A4 complex. M39 is shown in orange; subunits of S100A4 dimer are in green and cyan. (B) Lowest-energy structure of M39/S100A4 complex in two orientations related by 90° rotation along the vertical axis. The boxed areas indicate sites A and B. (C) Structure of bound M39. Hydrophobic residues of M39 in direct contact with S100A4 are highlighted in magenta and labeled. (D) Hydrophobic contacts at site A (left) and B (right). For clarity only the helices H3 and H4 are shown as viewed from the back in the orientation shown in (B). Helices H3 and H4 are oriented in a similar way for both sites. (E) Superposition of the M39/S100A4 structure on the structures of (from left to right) TRTK-12/S100B (PDB 3IQQ), TRTK-12/S100A1 (PDB 2KBM), and RyRP12/S100A1 (PDB 2K2F). M39 is shown in light orange, S100A4 in green and cyan, S100B and S100A1 in gray, and TRTK-12 and RyRP12 in red and magenta. Note that in contrast to M39/S100A4, each of these complexes is symmetrical and has a 2:2 configuration. Despite this difference, they match some features of the M39/S100A4 complex. TRTK-12 binding to S100B mostly resembles that of the C-terminal nonhelical portion of M39 and the last turn of the helix. TRTK-12 in complex with S100A1 aligns well with the N- and C-terminal ends of the M39 helix. The helix of RyRP12 in the complex with S100A1 is longer and extends further toward the adjacent EF2 site. See Figure S2 for further structural analysis and comparison with other S100 protein complexes.

The superposition of the lowest-energy structures is shown in Figure 2A, and the statistics of the structure calculation are summarized in Table 1. Both S100A4 and M39 are well defined in the calculated structure (backbone rmsd 0.50 and 0.54 Ǻ, respectively), corresponding to the large number of the intra- and intermolecular NOE restraints used. The myosin peptide forms a helix that is positioned across the EF hands of both subunits, connecting the two hydrophobic EF2-binding sites that become exposed on Ca^2+^ binding to S100A4 (Vallely et al., 2002; Gingras et al., 2008; Malashkevich et al., 2008) (Figure 2B). The hydrophobic residues located on one face of the amphipathic myosin helix (Figure 2C) make extensive contacts with the surface residues of S100A4. These contacts are particularly prominent at the hydrophobic pockets between the helices H3 and H4 (Figures 2B and 2D).

The two EF2 sites interact with the opposite ends of the M39 helix, making them structurally distinct and causing doubling of the NMRs. For simplicity of description we refer to the sites as A and B according to their contacts with, respectively, the N- or C-terminal regions of myosin. At site A the side chain of the N-terminal L1900 is inserted into a hydrophobic pocket formed by the side chains of L58, F78, I82, and M85, with additional contacts from Me groups of A1903 and T1904. At the site B the same hydrophobic residues of a different S100A4 subunit make contacts with the side chains of L1921 and L1926 at the C-terminal part of the M39 helix, as well as F1928 and V1930 from the nonhelical part of the molecule. The C-terminal part of M39 extends further along the groove between the helices toward the opposite face of the molecule, making additional hydrophobic contacts that involve P1931 and M1934. A close contact between M39 and S100A4 is supported by a large number of intermolecular NOEs detected in the complex. Figure S2A illustrates the NOEs for the Me groups of I82 that show a clear distinction between the contacts at site A and B.

In addition to the hydrophobic contacts, the charged and polar residues of M39 are optimally positioned for forming hydrogen bonds and salt bridges with the complementary S100A4 residues (Figure S2B). At the N terminus of M39, E1901 and E1905 are close to K57 and N61 of S100A4. In the mid-region N1911 and R1912 are in proximity to E74, whereas E1913 is close to N73. At the C-terminal part of the M39 helix, K1918 is close to N73, R1922 is near E88, and D1925 close to K57 and N61. The matching charges are likely to contribute to the energy of the interaction and enhance the selectivity of S100A4 toward the specific myosin isoform, NMIIA.

In the longer M70 fragment, the NOE pattern and chemical shift values indicate the formation of an additional helix at the N terminus (Figures S2E–S2G). No contacts were detected between this helix and S100A4, in agreement with increased dynamics in this region (Figure 1B). The N-terminal helix is separated from the S100A4-binding site by the unstructured region. The shorter M32 peptide (residues 1,907–1,938) binds with an affinity of 3 μM, which is sufficiently high to reach saturation at the concentrations used. However, the NMR spectra show only one set of signals for S100A4 at all peptide concentrations (Badyal et al., 2011). Some of these signals remain broadened even at high excess of M32, indicating exchange process within the complex. For M32 in the complex, we detected similar intermolecular NOEs as for the corresponding region of M39, demonstrating similarity of the interaction. Modeling of M32 on the structure of M39 complex shows that the peptide would extend into the second EF2 site and interfere with binding of the second peptide molecule. At the same time the peptide is not long enough to interact with both EF2 sites. As a result one site would either remain unoccupied, or binding of the second molecule would destabilize the complex. The exchange of the peptide between the sites enhanced by this self-competition would lead to the observed resonance broadening. A similar resonance broadening was reported for an even shorter peptide 1,908–1,923 (Malashkevich et al., 2008).

The conformation of S100A4 in the complex is similar to that of the Ca^2+^ form (PDB 3C1V; rmsd 1.6 Å;, Figure S2C). This demonstrates a limited effect of myosin binding on the S100A4 structure. Similar overall structure conservation was observed for complexes of other S100 proteins (Figures 2E and S2D) (Réty et al., 1999; Rustandi et al., 2000; Bhattacharya et al., 2003; Lee et al., 2008; Wright et al., 2008, 2009; Charpentier et al., 2010).

Simultaneous engagement of both EF2 sites of the S100A4 dimer in the binding of a single myosin molecule in an asymmetrical 1:2 configuration is novel for S100 proteins. In all other reported structures of S100 complexes (Réty et al., 1999; Rustandi et al., 2000; Bhattacharya et al., 2003; Lee et al., 2008; Wright et al., 2008, 2009; Charpentier et al., 2010), the EF2 sites on each monomer are occupied by independent ligand molecules in a symmetrical 2:2 complex (Figures 2E and S2D). Involvement of the two EF2 sites in S100A4 dramatically extends the binding interface, leading to nanomolar affinity not observed in any other S100 complexes. The location of the EF2-binding sites on one face of the molecule allows them to accommodate an uninterrupted long helix.

Asymmetrical binding has recently been reported for the interaction of AHNAK with a preformed S100A10/annexin complex (Rezvanpour et al., 2011). To our knowledge, the structure of the complex has not been solved yet, but the reported information suggests that the configuration of the complex is likely to be different from M39/S100A4. The AHNAK fragment of 18 residues is too short to link the 2 EF2 sites, and the NMR-binding site mapping indicates that the interactions are primarily restricted to the helices H4/H4′ of S100A10.

Similar to M39, all other reported S100 ligands adopt helical structures, although the orientations of the helices differ significantly between the complexes (Figure S2D). In most other ligands the helices are much shorter than M39 and can interact with only one EF2 site. One exception is SIP interaction with S100A6 (2JTT; Lee et al., 2008), where the ligand forms two helices. However, only the N-terminal helix binds to EF2 site, whereas the C-terminal region interacts with the Ca^2+^-independent site along the helix H1. This leads to a symmetrical 2:2-binding mode, distinctly different from that of M39/S100A4 complex.

Despite the differences in the overall binding mode, different regions of the N and C termini of the M39 structure in the complex with S100A4 superimpose well with the CapZ peptide (TRTK-12) in complex with S100B (Charpentier et al., 2010) and S100A1 (Wright et al., 2009) (PDB 3IQQ and 2KBM, respectively), as well as ryanodine (RyRP12) in complex with S100A1 (Wright et al., 2008) (PDB 2K2F), as illustrated in Figure 2E. This suggests that other S100 proteins may adopt the asymmetrical binding mode with suitable ligands or may interact with NMII with significant affinity.

### Effect of S100A4 on NMIIA in Cells

**Figure 3:**
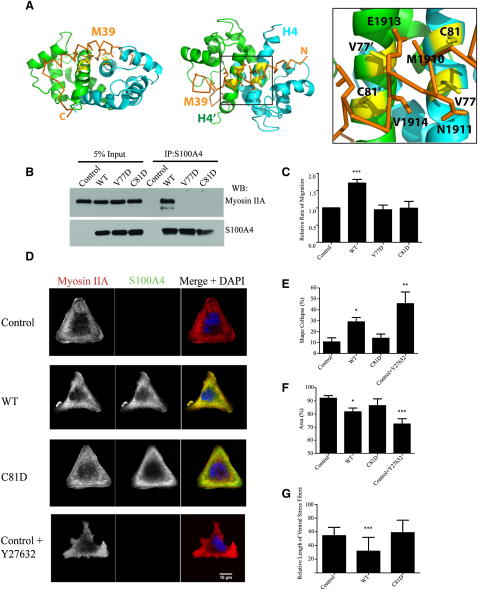
S100A4 Affects Stress Fibers, Shape, Spreading, and Motility of A431 Cells (A) Cartoon representation of the M39/S100A4 complex illustrating positions of the mutated residues. The two orientations differ by a 90° rotation along the horizontal axis. Zoomed region shows contacts of the mutated residues V77 and C81 (side chains are shown as sticks; residues are highlighted in yellow). M39 is shown in orange; side chains contacting V77 and C81 in the complex are presented as sticks and labeled. (B) V77D and C81D mutants have impaired interaction with NMIIA. S100A4-containing complexes were immunoprecipitated from lysates of nucleofected cells. Myosin IIA and S100A4 were detected by western blotting. As a control for loading, 5% of the input lysates were run on the gel. See Figure S3 for further characterization of binding of mutant S100A4. (C) WT but not mutant S100A4 activates cell migration in transwell assay. Migration rates were expressed as the ratio of the averaged number of migrating cells relative to the control that was set at 1. Values represent averages of three independent experiments each performed in triplicate. Error bars are SDs among three independent experiments. (D) Cell spreading on the collagen-coated micropatterned glass. Nucleofected cells, pBI empty vector (Control), or pBI vector expressing WT or S100A4 mutant (C81D) were seeded on the collagen type I micropatterned equilateral triangles (length, 41 μm). Cells were stained with antibodies against myosin IIA or S100A4. (E–G) Analyses were performed using images from 50 cells stained for myosin IIA and S100A4 as in (C). Error bars represent SDs between measurements of 50 cells. ^∗^p < 0.05 versus control; ^∗∗^p < 0.005 and ^∗∗∗^p < 0.0001. (E) Cell shape analysis. An area of a fully spread cell that adopted the maximum size of the collagen-coated equilateral triangle micropattern was set at 100% (see Figure S3). (F) Relative area analysis. The area of a spread cell was calculated as the percent relative to the triangle pattern area, 841 μm. (G) Comparative analysis of the peripheral stress fiber formation. Relative length of the stress fibers along the cells was measured and plotted against cell perimeter, where the perimeter was set at 100%.

Previous studies of the role of S100A4 in cell lines or animal models have exploited mutations that affected the Ca^2+^-binding properties, the dimerization interface, and C-terminal region (Kim and Helfman, 2003; Zhang et al., 2005; Ismail et al., 2008, 2010). Modifications of these regions change overall characteristics of S100A4 and its interactions with all ligands, making the mutations nonselective. Here, we have used structural information to design NMIIA-specific mutants. Despite extensive contacts (Figure 2), disruption of the S100A4/ NMIIA interaction while maintaining structural integrity and Ca^2+^-binding properties is not straightforward. Many NMIIA-contacting residues are buried in the apo form of S100A4, and their modification would destabilize the protein. However, the unique myosin-binding mode offers an unexpected solution. The middle of the M39 helix crosses the exposed surfaces of the two equivalent and antiparallel helices H4/H4′ from different subunits (Figure 3A). As a result, the side chains of V77/V77′ and C81/C81′ become buried under the hydrophobic surface of the M39 helix and make extensive hydrophobic interactions: V77 contacts A1907, M1910, and N1911; V77′ contacts E1913, V1914, and L1917; C81 contacts T1906, A1907, and M1910; and C81′ contacts V1914, L1917, and K1918. The change of either of these residues to Asp in V77D or C81D mutants would introduce two negatively charged groups from different subunits under the myosin hydrophobic surface and destabilize the complex.

In agreement with the prediction, the mutations strongly reduce the affinity, while having practically no effect on S100A4 structure and its interaction with Ca^2+^ (Figures S1I and S3A–S3D). NMR titration data (Figures S3C and S3D) show that the K_D_ values are ∼0.1 mM, corresponding to an increase by >10^5^ relative to the wild-type (WT). Because the mutations do not affect the EF2 sites directly but disrupt their simultaneous engagement, the large reduction in affinity on mutations demonstrates that the involvement of both sites is crucial for S100A4/NMIIA interaction. In agreement with our results, C81 was identified recently as a residue critical for the interaction with NMIIA using modification of the exposed Cys residues (Dulyaninova et al., 2011).

Having characterized the mutants in vitro, we explored the effect of these mutations in cells. Both V77D and C81D S100A4 were expressed in human epithelial carcinoma A431 cells (Andersen et al., 2005) at levels close to the WT (Figure S3E) but showed no interaction with the intact NMIIA in a coimmunoprecipitation assay (Figure 3B), similar to the in vitro results. Expression of the WT S100A4 promoted A431 cell migration in a transwell assay, but neither of the mutants produced any effect above the empty vector (Figure 3C). Because the migration rate is likely to reflect myosin filament stability, we sought an assay to demonstrate a direct effect of S100A4 on the structure of NMIIA filaments. Although we could observe an effect of S100A4 on cell morphology during spreading on uniform collagen or fibronectin surfaces, we were unable to measure it reliably because the cell populations were heterogeneous. We, therefore, used micropatterns as a more controlled way of attaching cells to surfaces. Spread cells acquire the defined micropattern shape with a similar distribution of the cytoskeletal structures within different cells (Picone et al., 2010; Théry, 2010).

A431 cells were plated on collagen equilateral triangles with 41 μm sides, yielding a total area of 841 μm^2^ (Figures 3D and S3G). This size enabled an A431 cell to spread entirely on an individual micropattern. Control cells or cells expressing S100A4 mutants acquired a triangular shape 90 min after plating. In contrast we observed a collapse of the defined cell borders in all cells expressing WT S100A4 (Figures 3D–3F, S3F, and S3G). The observed effect was similar to more extreme changes in cell phenotype produced by the Rho kinase inhibitor Y27632, a potent inducer of acto-myosin filament disassembly (Figures 3D–3F, S3F, and S3G) (Théry, 2010). In agreement with this observation, staining for NMIIA demonstrated the enrichment in myosin filaments at the periphery of triangularly shaped control or C81D mutant-expressing cells (Figure 3G). Here, the majority of the cells contained large ventral stress fibers marking the cell perimeter. However, cells overexpressing WT S100A4 showed a reduction in the number of stress fibers, and ventral stress fibers were either completely absent or less pronounced than in control or C81D mutant-expressing cells (Figures 3G and S3G). Previously, Li et al. (2010) reported that S100A4 mildly increased the presence of NMIIA in the Triton-soluble fraction of cell lysates prepared from CSF-1-treated macrophages. Our results are consistent with this observation and, to our knowledge, represent the first microscopic evidence that S100A4 reduces myosin filament assembly in vivo.

### S100A4 Interaction with Longer Myosin Fragments

In the whole myosin molecule, S100A4 interacts with the region initially assembled into the coiled-coil structure. This sequence has a high propensity to form a helical structure. However, M70 in the complex with S100A4 contains a clear break in the helical structure at the N terminus of the S100A4-binding site (Figure S2F). The rational for this is provided by the structure of the complex. In the complex the N-terminal end of the M39 helix is in close proximity with the H2-H3 loop of S100A4 that contains two positively charged residues K48 and R49 (Figure S2G). The continuation of the myosin helical structure at the N terminus in the M70 fragment would create a steric clash with the side chains of these residues. Additionally, the myosin sequence in this region ^1893^SRRKL^1897^ creates a high concentration of positive charges that repel K48 and R49. These two factors are expected to destabilize the myosin structure in the complex, leading to the detected unstructured hinge region. Further toward the N terminus of M70, the helical structure is reestablished, as expected from the sequence composition. This part of the molecule is extended beyond the S100A4-binding site and does not make any contacts with the protein.

**Figure 4:**
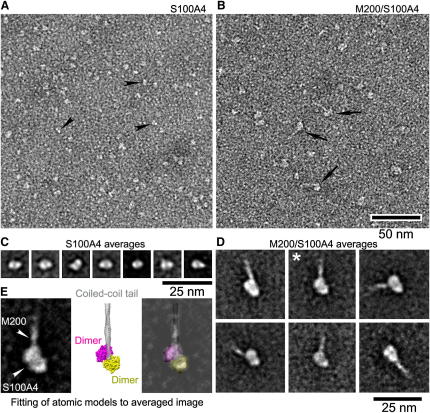
Effect of S100A4 on the Coiled-Coil Structure of Myosin (A and B) Negatively stained fields of S100A4 alone and M200/S100A4 complex. Black arrowheads and arrows point to individual molecules in each field. (C and D) Representative averaged images of S100A4 alone and M200/S100A4 complex, respectively. Each averaged image contains 30–50 images. (E) Fitting of S100A4 and coiled-coil tail atomic models to M200/S100A4 averaged image. Left panel is a selected averaged image of M200/S100A4 (taken from asterisk-marked average in D). Middle panel is an equivalent view of assembled atomic models to the average. Right panel is a superposition of equivalent view of the atomic model on the average. The lowest-energy structure of the complex was used to model M39/S100A4; coiled-coil myosin model was based on the structure of myosin V (PDB 2DFS). White arrowheads in the left panel indicate the region of S100A4 in M200/S100A4 and the emergence of the M200 densities from the S100A4. Scale bars apply to the field in (A) and (B) (50 nm) and averaged images of class images in (C) and (D) (25 nm).

The exposed helical region of M70 in the complex is not sufficient for the formation of the coiled-coil structure. However, the longer fragment M200 (Q1761–E1960) has the required characteristics and forms a stable-coiled coil structure (Badyal et al., 2011). We carried out single-particle analysis of negatively stained electron micrographs of the S100A4/M200 complex (Figure 4). At physiological ionic strength (100 mM Na acetate), free S100A4 forms dimeric structures, equivalent in size and shape to the dimeric crystal structure of S100A4, with a length of 5–6 nm and a width of 3–4 nm (Figures 4A and 4C). In the presence of M200, the images show a globular structure with a length of 9–10 nm and a width of 6–7 nm connected to a thinner rod (Figures 4D and 4E). Two-dimensional fitting to the average shape demonstrates that the globular structure has the dimensions corresponding to two S100A4/M39 complexes arranged in a close proximity, whereas the rod structure has the geometrical characteristics of the NMIIA coiled coil (Figure 4D) and is similar to that observed for free M200 (Badyal et al., 2011). This directly demonstrates association of two S100A4 dimers with the stable coiled-coil myosin fragment, in agreement with the stoichiometry of the complex determined above. The length of the coiled-coil region observed in the averaged image is less than expected for the M200, suggesting some flexibility of the coiled coil. The orientation of the globular part relative to the coiled coil shows some variation between the images, as expected from the disordered structure detected at the N terminus of the binding site (see above).

## Discussion

**Figure 5:**
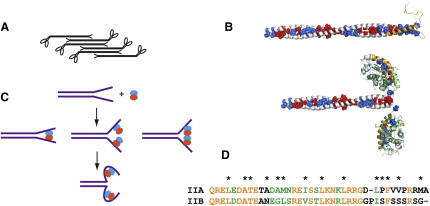
S100A4 Interaction with Myosin Filaments (A) Schematic diagram of the assembled filament illustrating staggered packing of myosin units. (B) Model of the C-terminal region of the myosin coiled coil (top) and myosin-S100A4 complex (bottom). Side-chain atoms of the residues in positions *a* and *d* of the heptad repeat that are crucial for the coiled-coil stability shown as spheres. Large hydrophobic residues that stabilize coiled coil are highlighted in blue; nonoptimal destabilizing residues are in red. S100A4-binding site in myosin is colored in orange and the rest of the myosin in gray. S100A4 subunits are shown in pale green and pale blue. A patch of nonoptimal residues precedes the S100A4-binding site. (C) Schematic diagram of the S100A4 interaction with myosin showing possible intermediates. Subunits of the S100A4 dimer are shown in red and blue. Initially, S100A4 may interact with the unstructured region that is likely to be accessible in the filaments. These sites could engage separate S100A4 dimers or simultaneously bind to the two EF2 sites of a single dimer. The binding of the two dimers to the unstructured region of the coiled coil could be further stabilized by the tetramer formation detected in the crystal structure of S100A4 (Gingras et al., 2008). Potentially, all three intermediate states could exist in equilibrium. (D) Sequence comparison of the S100A4-binding site in NMAIIA with the corresponding region in NMAIIB. The residues involved in the direct contact with S100A4 are marked with an asterisk (“^∗^”). Identical residues are highlighted in orange, similar in green. Chemically distinct residues are shown in black.

The asymmetrical 1:2 ligand-binding mode identified here for the NMIIA/S100A4 complex has strong biological implications. NMII filaments are formed as the result of staggered packing of the coiled-coil regions against each other (Figure 5A) stabilized by the matched distribution of positive and negative charges (McLachlan and Karn, 1982; Atkinson and Stewart, 1992; Ricketson et al., 2010). In vitro studies indicate that S100A4 can actively dissociate myosin filaments, rather than just sequester myosin monomers (Badyal et al., 2011). In order to disassemble filaments, S100A4 interaction with NMII has to meet specific conditions: (1) high affinity of the interaction; (2) accessibility of the binding site in the filament; and (3) disruption of the packing in the filament. The extensive binding interface of the S100A4/NMIIA complex favors all three conditions.

Our quantitative analysis (Badyal et al., 2011) argued that filament disassembly requires nanomolar affinity for the interaction of S100A4 with the monomeric myosin. In the present work we show that the required affinity originates from the simultaneous contribution (Jencks, 1981) of the EF2 sites from both monomers of the S100A4 dimer into myosin binding. Notably, most other interactions reported for S100 proteins involve a single Ca^2+^-dependent EF2 site from one subunit and have affinities in the micromolar range. An apparent exception is the interaction of S100A10 with AHNAK that has a K_D_ of 167 nM (Rezvanpour et al., 2011). However, AHNAK only binds to S100A10 that is already in complex with annexin and is likely to represent a different configuration of the complex (see above). The data on the range of S100 complexes suggest that a limited number of contacts at a single site are not sufficient to generate high-binding energy required for the disassembly of NMII filaments. Our conclusion is supported by the recent study of the fused S100A4 dimer where one binding site was compromised by mutations (House et al., 2011).

The K_D_ of ∼1 nM evaluated here for M39 by a range of methods is significantly lower than reported for both smaller and larger NMIIA fragments (Ismail et al., 2008; Malashkevich et al., 2008). Smaller myosin fragments do not include the full S100A4-binding site and, therefore, have lower affinity (micromolar to millimolar; Badyal et al., 2011). Longer myosin fragments form coiled coils, and therefore, part of the binding energy of S100A4 is used to separate the strands. Furthermore, at physiological ionic strength, longer rod fragments (>100 residues) form filamentous aggregates, and this interaction has to be overcome for S100A4 to bind to the monomeric unit. Hence, although longer fragments contain the complete binding site, the measured binding constant can be much weaker than that we observe for M39 (e.g., in the micromolar region for the intact myosin rod; Ford et al., 1997; Li et al., 2003).

Packing of the NMII filament strongly reduces access of other proteins to the coiled-coil region. Additionally, the residues from the coiled-coil part of the binding site that contact S100A4 are also buried in the coiled-coil interface (Figure 5B). However, the unstructured C-terminal region of NMIIA is expected to remain unstructured and dynamic even in the fully formed NMIIA filament. This region may provide the initial docking site for S100A4, with the docking facilitated by the proximity of two S100A4-binding sites in the adjacent strands of the coiled coil. This suggests a model where the binding proceeds through an intermediate state in which S100A4 initially interacts with the unstructured region (Figure 5C). The individual chain of the coiled-coil part of NMIIA that interacts with S100A4 in the complex retains its helical structure, and all the hydrophobic residues that initially formed the coiled-coil interface make contact with S100A4. We propose an unzipping mechanism that can facilitate the complex formation. In this mechanism, S100A4 initially docks weakly to the unstructured region and then “rolls over” the hydrophobic face of the myosin helix, gradually engaging the rest of the binding site residues, thereby increasing affinity. In this way the myosin hydrophobic residues are not exposed to bulk water in the intermediate state, reducing the entropic barrier of the transition. Immediately upstream of the S100A4-binding site, the NMIIA sequence contains residues that destabilize the coiled coil (Figure 5B). This is likely to favor the full transition into the complex by creating a natural break in the coiled coil.

Additionally, the myosin helix is destabilized upstream to the binding site, creating a disorder in the S100A4 orientation relative to the myosin coiled coil. This disorder is detected by EM, where distinctly different arrangements are observed (Figure 4D). Binding of the independent S100A4 dimer to each chain of the myosin coiled coil increases the disorder in this region and creates a large bulge that would disrupt the filament packing.

The high affinity of monomeric NMIIA for S100A4 in the presence of Ca^2+^ and the high affinity of the NMIIA-S100A4 for Ca^2+^ (required for thermodynamic balance) imply that these proteins could interact even at resting cytoplasmic Ca^2+^ concentrations (Badyal et al., 2011). However, interactions would be stronger under conditions of excitation that may be localized within specific regions of a cell. This property, along with potential cross-reactivity and degeneracy between both NM and S100 isoforms, complicates investigation of interactions in vivo. We tested a wide range of support materials for migration and spreading assays and found that the degree of the effect of S100A4 expression was strongly dependent on the substrate type and density; with high-density collagen giving clearest effect. Additionally, differences in spreading on collagen micropatterns in the presence of S100A4 or the binding-deficient C81D mutant was time dependent (Figure 3). Nevertheless, A431 cells expressing WT S100A4 consistently showed a reduced number of stress fibers. We consider that S100A4 becomes involved in the myosin regulation under specific conditions when the Ca^2+^ concentration and filament stability are within the optimal range for the interaction. The observed substrate dependence suggests an interesting possibility that S100A4 contributes to the feedback mechanism involved in the detection of the substrate stiffness. If valid this hypothesis has an implication for the role of S100A4 in metastasis.

Comparison of NMIIA and IIB sequences shows that the selectivity of S100A4 for NMIIA is associated with the differences in the C-terminal part of the binding site (Figure 5D). Hydrophobic residues F1928, V1930, and M1934 that make contacts with S100A4 are replaced by hydrophilic serine residues in NMIIB. These and neighboring serine residues in NMIIB are phosphorylated by α-kinases TRMP6 and TRMP7 (Clark et al., 2008), which would further reduce the interaction with S100A4. The residues in the coiled-coil region are very similar in the two isoforms and are expected to make comparable contributions into the binding. Notably, the previously suggested minimal binding site 1,908–1,923 (Malashkevich et al., 2008) does not include the unstructured region and cannot readily explain the isoform selectivity of S100A4.

In summary, our structure of the S100A4/NMIIA complex shows an asymmetrical binding mode that simultaneously involves both EF2 sites of the S100A4 dimer. This generates a high affinity for NMIIA with the K_D_ below 1 nM, unique in the S100 family of proteins. In the case of S100A4, such a high affinity is required for the disassembly of myosin filaments. For the nonfilamentous targets of S100 proteins, nanomolar affinity may be biologically inappropriate. It remains to be seen if the S100A4-binding mode has evolved uniquely to match the properties of NMIIA filaments.

After acceptance of this paper, we became aware of the results of Kiss et al. (2012), who also investigated the structure and interaction of a myosin peptide with S100A4 and reached overall similar conclusions. In order to crystallize the complex, they introduced four mutations in S100A4, but the conformation of the bound myosin peptide (PDB 3ZWH) showed only local differences around the sites of these mutations compared with our NMR structure.

## Experimental Procedures

### NMR Spectroscopy

For the structural analysis, samples contained 0.8–1 mM of S100A4 in complex with either M39 or M70 at the stoichiometry 1:2 S100A4:peptide in the following combinations: u-M39/^13^C,^15^N-S100A4, u-M70/^13^C,^15^N-S100A4, and ^13^C,^15^N -M70/u-S100A4. The buffer contained 20 mM 4-morpholineethanesulfonic acid (pH 6.1), 20 mM NaCl, 5 mM CaCl_2_, 4 mM TCEP, 5% (v/v) of ^2^H_2_O. Spectra used for the resonance assignments and structure calculations are detailed in the Supplemental Experimental Procedures.

### NMR Structure Calculations

The structure of the complex was calculated with Aria 1.2 in two stages, as fully described in the Supplemental Experimental Procedures. Briefly, the first stage was used to generate initial docking model from a subset of intermolecular NOEs with the assignments that can be validated in all the filtered experiments collected for u-M39/^13^C,^15^N-S100A4, u-M70/^13^C,^15^N-S100A4, and ^13^C,^15^N-M70/u-S100A4. In the second stage all intermolecular NOEs assigned independently were used. In addition all intramolecular NOEs were used at both stages. For S100A4, intramolecular NOEs were derived from the 3D NOESY-HSQC spectra of u-M39/^13^C,^15^N-S100A4, for M39 from 3D NOESY-HSQC spectra of ^13^C,^15^N-M70/u-S100A4, and 2D filtered NOESY experiment measured for u-M39/^13^C,^15^N-S100A4. Throughout the calculation dihedral angle restraints and symmetry restraints for N-terminal half of S100A4 (residues 1–44) were applied. Finally, 200 structures were calculated at each Aria iteration, 30 best refined in the presence of explicit water molecules, 20 lowest-energy structures used for the analysis and PDB deposition. Statistics of the structure calculation are presented in Table 1. Ramachandran statistics for the final ensemble of structures shows that 84.5% of residues are in the most favorable, 14.4% in allowed, 0.6% in generally allowed, and 0.5% are in disallowed regions.

### Immunoblot Analysis

Cells were lysed in NP-40 buffer (150 mM NaCl, 50 mM Tris [pH 8.0], 0.5% NP-40, 1 mM dithiothreitol, 10 mM α-glycerophosphate, 50 mM NaF, 0.1 mM Na_3_VO_4,_ 2 μg/ml aprotinin, 10 μg/ml leupeptin, 1 mM PMSF) for 10 min on ice followed by centrifugation. For immunoprecipitation, proteins (500 μg) were incubated with 5 μg of anti-S100A4 Mab (DSHB University of Iowa, Iowa City, IA, USA) overnight. Beads were washed four times, S100A4 complexes were eluted in gel loading buffer and resolved on 6% and 15% SDS-PAGE gels. Proteins were transferred to PVDF membranes (Millipore) and stained for S100A4 (Dako, Denmark) or heavy chain of NMIIA (Covance, Princeton, NJ, USA). To control for protein expression levels, 5% of the input supernatant was used.

### Cell Culture and Assays

A clone of human epithelial carcinoma A431 cells (Andersen et al., 2005) was used throughout the study. Both V77D and C81D S100A4 were expressed in S100A4-negative human epithelial carcinoma A431 cells (Andersen et al., 2005). Cell migration was analyzed in a transwell assay according to a standard protocol with minor modifications (Andersen et al., 2005). Full details are in the Supplemental Experimental Procedures.

### Immunofluorescence Analysis

Cells were cultured on coverslips, rinsed with PBS, fixed in 4% paraformaldehyde for 20 min, and permeabilized in 0.5% Triton X-100 for 5 min. Primary antibodies were anti-S100A4 Mab (DSHB University of Iowa) and polyclonal anti-heavy chain of NMIIA (Covance). Slides were stained with secondary anti-mouse 488 Alexa or anti-rabbit 568 Alexa (Invitrogen, Paisley, UK) antibodies. All antibodies were diluted in DMEM containing 10% FBS. After counterstaining with DAPI (Molecular Probes), cells were examined and photographed using a Nikon C1Si confocal laser-scanning microscope equipped with a CFI Plan Apo 60x, 1.4NA objective lens.

### Cell Patterning

Micropatterning, master, and PDMS stamp fabrication were carried out as described previously (Picone et al., 2010). Micropattern printing is described in the Supplemental Experimental Procedures. Seventy-two hours after nucleofection, cells were lifted with trypsin (Trypsin-EDTA, PAA), resuspended first in a small amount of medium with 10% FBS and then diluted in serum-free medium to a density of 2.5 × 10^5^/ml. A total of 2 ml of suspension was used for seeding on the micropatterned glass surface. Typically, cells attached to the printed patterns within 10 min of seeding. Nonadherent cells were removed by double washes with PBS. Cells were allowed to attach and spread for 90 min before fixation. For experiments with ROCK inhibitor Y27632 (Sigma-Aldrich, St. Louis), the inhibitor was added 15 min prior to fixation at a final concentration of 10 μM. Immunofluorescence analysis was performed as described above. Micropatterns were stained with anti-collagen type I (clone COL-1) antibody from Sigma-Aldrich, UK. Image and statistical analyses are described in the Supplemental Experimental Procedures.

### EM and Single-Particle Analysis

For negative staining, purified S100A4 protein was diluted with 100 mM Na acetate, 0.1 mM CaCl_2_, 3 mM MgCl_2_, 10 mM imidazole, 5 mM NaH_2_PO_4_ (pH 7.0) (room temperature) to give a final concentration of 400 nM. For visualizing its complex with M200 fragments, purified S100A4 (1μM) and M200 (1 μM) were mixed with the aforementioned dilution buffer then crosslinked by 0.1% glutaraldehyde for 1 min. The resulting mixture was further diluted 5-fold with the same dilution solution to give a final concentration of 200 nM of each. A total of 5 μl of each sample preparation was applied to a carbon-coated grid that had been glow discharged (Harrick Plasma, Ithaca, NY, USA) for 3 min in air, and the grid was immediately negatively stained using 1% uranyl acetate (Jung et al., 2008). Grids were examined in a Philips CM120 (FEI, Hillsboro, OR, USA) operated at 80kV, and images were recorded on a 2K×2K F224HD SlowScan CCD camera (TVIPS, Berlin). For single-particle analysis, images of individual particles on micrographs were selected interactively, windowed out, and imported into the SPIDER program suite (Health Research, Rensselaer, NY, USA). Of the particles, 959 (S100A4) and 919 (M200/S100A4 complex) were used in the processing, and class averages were produced by a reference-free method (Burgess et al., 2004). UCSF Chimera was used for visualization and comparative analysis between atomic models and averages (Pettersen et al., 2004).

## Figures and Tables

**Table 1:** NMR and Refinement Statistics for M39/S100A4 Complex

NMR Distance and Dihedral Constraints	M39/S100A4
Distance constraints	
Total NOE	7,085
Intra-residue	377 (M39), 2,071 (S100A4)
Inter-residue	
Sequential (|*i* − *j*| = 1)	389 (M39), 1,634 (S100A4)
Medium range (|*i* − *j*| < 4)	326 (M39), 1,356 (S100A4)
Long range (|*i* − *j*| > 5)	1,783 (S100A4)
Intermolecular	977
Hydrogen bonds	0
Total dihedral angle restraints	
ϕ	156 (S100A4), 26 (M39)
ψ	156 (S100A4), 26 (M39)

**Structure Statistics**	

Violations (mean ± SD)	
Distance constraints (Å)	0.05 ± 0.004
Dihedral angle constraints (°)	1.9 ± 0.3
Maximum dihedral angle violation (°)	8
Maximum distance constraint violation (Å)	0.5
Deviations from idealized geometry	
Bond lengths (Å)	0.005 ± 0.00014
Bond angles (°)	0.72 ± 0.017
Impropers (°)	2.1 ± 0.14
Average rmsd to mean structure (Å)^a^	
Heavy	0.91
Backbone	0.50

a Rmsd was calculated among 20 refined structures.

## Accession Numbers

The coordinates for the structure of M39/S100A4 complex and chemical shifts have been deposited to PDB, accession code 2LNK. Chemical shifts have been deposited BRMB accession code 18169.

## Supplemental Information

Document S1. Figures S1–S3, Table S1, and Supplemental Experimental Procedures
