# Supplementary material for: Asymmetric Mode of Ca2+-S100A4 Interaction with Nonmuscle Myosin IIA Generates Nanomolar Affinity Required for Filament Remodeling
Source: Structure. 2012 Apr 4;20(4-2):654–66. doi: 10.1016/j.str.2012.02.002 (PMC3343272; doi:10.1016/j.str.2012.02.002)
Supplement: Document S1. Figures S1–S3, Table S1, and Supplemental Experimental Procedures [file mmc1.pdf]

Structure, Volume *20*

Supplemental Information

Asymmetric Mode of Ca^{2+}-S100A4 Interaction with Nonmuscle Myosin IIA Generates Nanomolar Affinity Required for Filament Remodeling

Paul R. Elliott, Andrew F. Irvine, Hyun Suk Jung, Kaeko Tozawa, Martyna W. Pastok, Remigio Picone, Sandip K. Badyal, Jaswir Basran, Philip S. Rudland, Roger Barraclough, Lu-Yun Lian, Clive R. Bagshaw, Marina Kriajevska, and Igor L. Barsukov

Inventory of Supplemental Information

Figure S1, related to Figure 1, presents additional data on S100A5 interaction with myosin fragments. This information is supports stoichiometry and affinity of the interaction.

Figure S2, related to Figure 2, provides additional information on the structural features of M39/S100A4 complex and presents comprehensive comparison of M39/S100A4 with other complexes of S100A4 proteins

Figure S3, related to Figure 3, provides validation of the dimeric form and folding of the S100A4 mutants, and cell images that illustrate the analysis of the cell shape and variations across the cells.

Table S1, related to Figure 1, summarises molecular weight measurements by SEC-MALLS

Supplementary methods provide details of the experiments

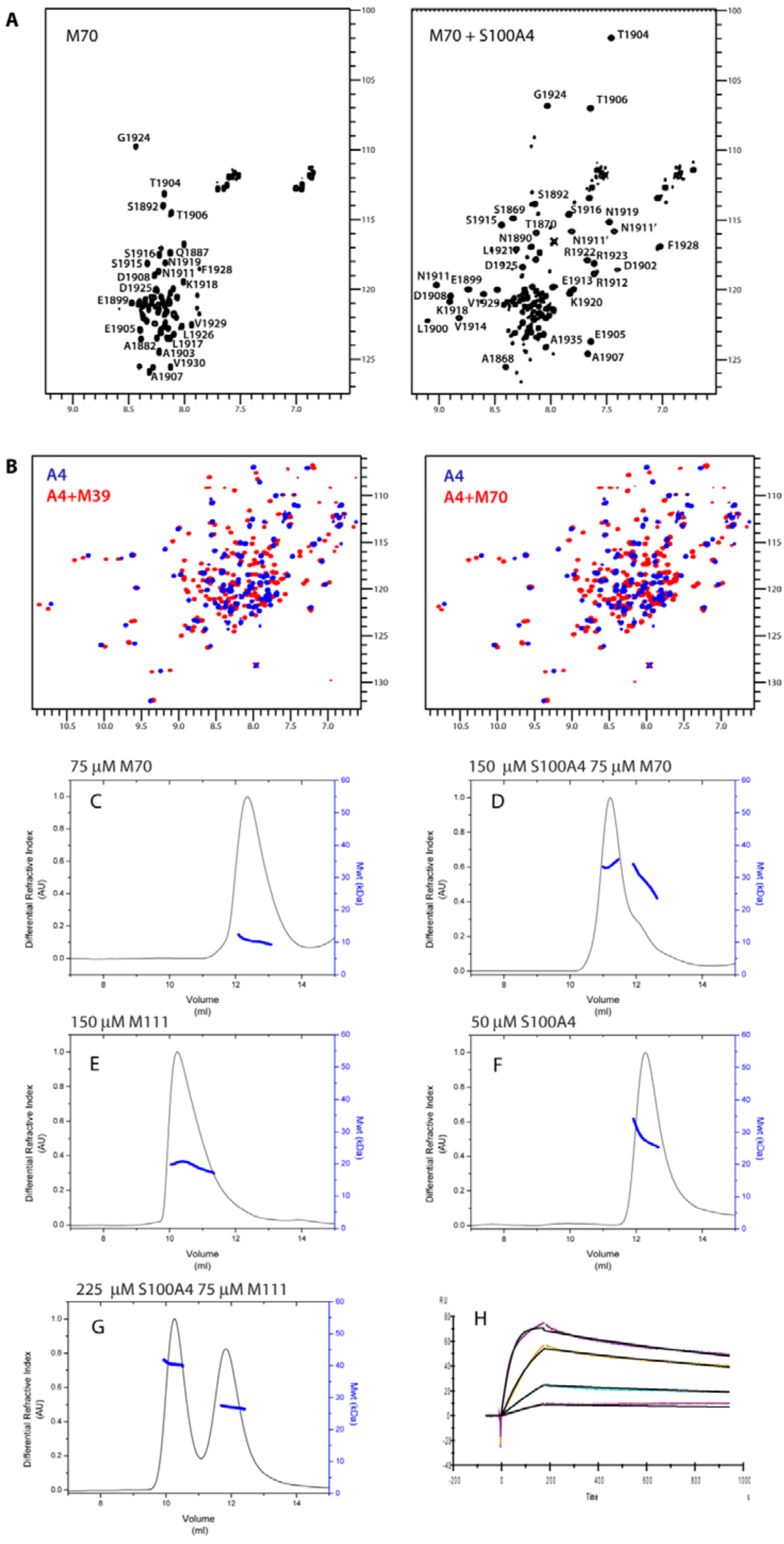
A
M70
M70 + S100A4
G1924
T1904
S1892
T1906
S1916
Q1887
S1915
N1919
D1908
N1911
F1928
D1925
K1918
E1899
V1929
E1905
L1926
A1882
L1917
A1903
V1930
A1907
T1906
S1869
S1915
T1870
N1890
N1911'
L1921
R1922
R1923
D1925
D1902
E1913
R1912
E1899
D1908
V1929
K1920
K1918
L1900
V1914
A1935
A1868
B
A4
A4+M39
A4+M70
75 μM M70
150 μM S100A4 75 μM M70
150 μM M111
50 μM S100A4
225 μM S100A4 75 μM M111
C
D
E
F
G
H
Differential Refractive Index (AU)
Volume (ml)
Mwt (kDa)
RU
Time
s

(I)

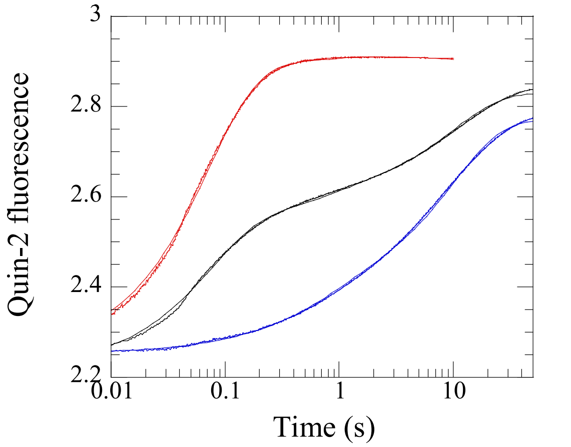
Quin-2 fluorescence
3
2.8
2.6
2.4
2.2
0.01
0.1
1
10
Time (s)

(J)

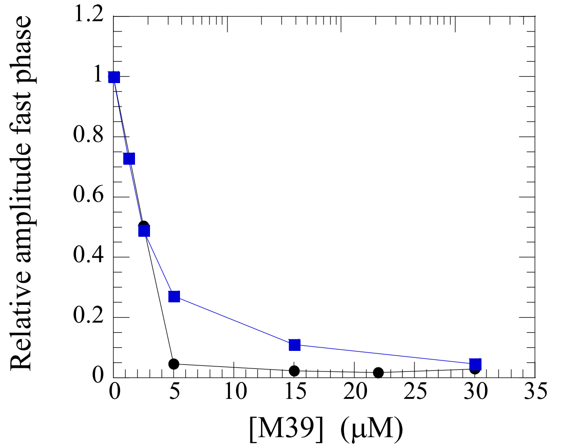
Relative amplitude fast phase
1.2
1
0.8
0.6
0.4
0.2
0
0
5
10
15
20
25
30
35
[M39] (μM)

(K)

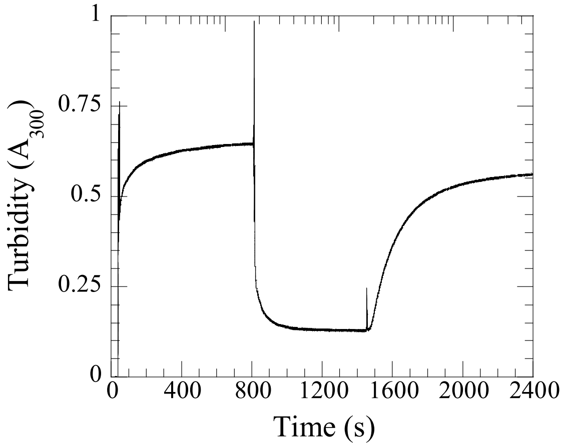
Turbidity (A_{300})
1
0.75
0.5
0.25
0
0
400
800
1200
1600
2000
2400
Time (s)

(L)

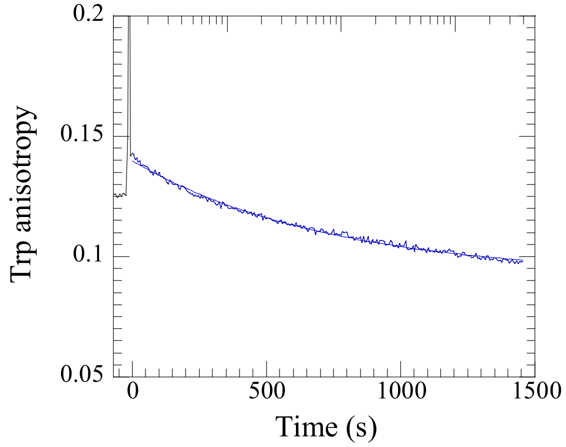
Trp anisotropy
0.2
0.15
0.1
0.05
0
500
1000
1500
Time (s)

(M)

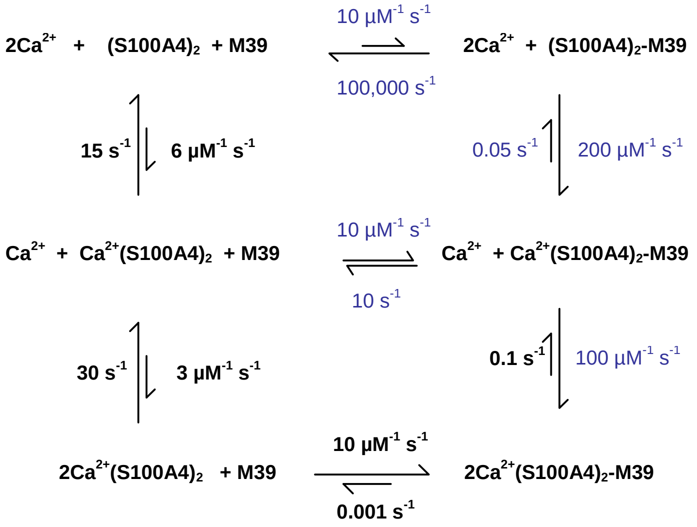
$2Ca^{2+}$ + $(S100A4)_2$ + M39
10 μM^{-1} s^{-1}
100,000 s^{-1}
$2Ca^{2+}$ + $(S100A4)_2$-M39
15 s^{-1}
6 μM^{-1} s^{-1}
0.05 s^{-1}
200 μM^{-1} s^{-1}
Ca^{2+} + $Ca^{2+}(S100A4)_2$ + M39
10 μM^{-1} s^{-1}
10 s^{-1}
Ca^{2+} + $Ca^{2+}(S100A4)_2$-M39
30 s^{-1}
3 μM^{-1} s^{-1}
0.1 s^{-1}
100 μM^{-1} s^{-1}
$2Ca^{2+}(S100A4)_2$ + M39
10 μM^{-1} s^{-1}
0.001 s^{-1}
$2Ca^{2+}(S100A4)_2$-M39

Figure S1, related to Figure 1. Interaction of S100A4 with myosin fragments.

(**A**) ^{15}N-HSQC spectra of ^{15}N-M70 in the free form and in complex with u-S100A4. Low intensity sharp signals are due to the presence of a small amount of degraded M70 that does not interact with S100A4. Assignments of sufficiently resolved resonances are indicated.

(**B**) ^{15}N-HSQC spectra of ^{15}N-S100A4 in the free form (blue) and in complex with u-M39 (red, left) and u-M70 (red, right). Note similarity of the chemical shift changes on the binding of these two myosin fragments, reflecting similarity of the interactions.

(**C-G**) SEC-MALLS elution profiles and molecular weight distributions. Molecular weights determined by MALLS in combination with refractive index and absorbance detection are summarised in Supplementary Table 1. Note that these measurements result is absolute molecular mass that is independent of the SEC elution profile and allow the protein concentrations to be determined. All measurements agree with 1:2 stoichiometry of the complex. Free M111 and M70 are eluted as broad peaks corresponding to a mixture between dimeric and monomeric forms. The fraction of the dimeric form is higher for M111. Note that peak corresponding to M111/S100A4 complex is well resolved from the peak of the free S100A4, but overlaps with the peak corresponding to free M111. To ensure full conversion into the complex a 1:3 M111:S100A4 molar ratiowas used to measure the molecular weight. At this ratio M111/ S100A4 complex and free S100A4 are present. For M70 both free forms are eluted close to the M70/S100A4 complex.The molecular weight of the complex was measured just above 1:2 M70:S100A4 molar ratio where a small amount of free S100A4 is detected as a shoulder.

(**H**) SPR sensograms of the S100A4 interaction with M39. The curves correspond to 1, 3, 9 and 27 nM of M39. Black curves represent fitting to 1:1 Langmuir model with K_d = 0.4 nM.

(I) Effect of M39 peptide on the dissociation of Ca^{2+} from S100A4. Ca^{2+} dissociation was monitored by Quin-2 fluorescence using a stopped-flow fluorimeter. One syringe contained 13 μM S100A4 (monomer concentration)), 50 μM Ca^{2+} and the other syringe 100 μM Quin-2 (reaction chamber concentrations). Both syringes contained 20 mM NaCl, 10 mM Hepes, 1 mM $MgCl_2$ at pH 7.5 and 20 ^{o}C. Records are shown for 0 (red), 2.5 (black) and 5 μM (blue) M39 premixed with the S100A4. Traces were analysed initially by fitting to a biphasic exponential to determine the rate constants of Ca^{2+} dissociation from the free S100A4 (14 s^{-1}), the putative contaminating tetrameric S100A4 (1.5 s^{-1}) and the M39-S100A4 complex (0.1 s^{-1}). The traces were then re-analysed by fitting a triphasic exponetial with rate constants fixed at these values and with variable amplitudes to determine the contribution of each of these species (i.e. the analysis assumes the M39 dissociates slowly compared with Ca^{2+} as indicated in panel (D)). The contaminating putative tetrameric species (see (Badyal et al., 2011)) remained below 20% throughout. The corresponding stopped-flow measurements with the V77D and C81D S100A4 mutants showed similar Ca^{2+} dissociation rate constants (14 s^{-1} and 8 s^{-1} respectively) compared to WT S100A4, but these were unaffected by addition of up to 50 μM M39, indicating the Ca^{2+} site was little changed, but M39 binding was much weaker.

(J) Analysis of the stopped-flow records as illustrated in (I) to determine the fraction of free S100A4 with increasing [M39]. Traces were fitted to a triphasic exponential and the amplitude of the fast phase (14 s^{-1}) was plotted against [M39]. Stopped-flow experiments were carried out with either the M39 premixed with the S100A4 (black circles) as described in (I) or M39 was in the same syringe as the Quin-2 (blue squares). The shape of the first titration (black line) indicates stoichiometric binding of 1 M39 to 2.6 S100A4 monomers with a K_d < 0.5 μM. The second titration (blue line) provides a measure of the M39 association rate constant to the Ca^{2+}-S100A4 because the amplitude of the fast phase represents the competition between Ca^{2+} dissociation from Ca^{2+}-S100A4 (14 s^{-1}) and M39 binding to it and trapping the Ca^{2+}. Modelling these data indicate the association rate constant for M39 to Ca^{2+}-S100A4 is ≈ 10^7 $M^{-1}.s^{-1}$.

(K) Competitive turbidity assay to determine the K_d for M39 binding to S100A4. 12.5 μM M200 was diluted from a high ionic strength buffer into 100 mM NaCl, 20 mM Hepes, 1 mM Mg^{2+}, 100 μM Ca^{2+} and filament formation was monitored by the turbidity at 300 nm. At 800 s, 25 μM S100A4 (monomer) was added to dissociate the filaments (cf. (Badyal et al., 2011)) . At 1450 s, 10 μM M39 was added which preferentially bound to the S100A4 and displaced >

70% of the M200 from the M200.S100A4 complex which then reformed M200 filaments. A previous assay using the M32 peptide as a competitor required 250 μM M32 to effect 50% dissociation of the M200 filaments (Badyal et al., 2011). As the K_d for M32 binding = 3 μM, these data indicate the K_d for M39 binding to S100A4 in the presence of Ca^{2+} < 0.01 μM.

(L) Dissociation of M39 from S100A4 in the presence of 100 μM Ca^{2+}. Typtophan fluorescence anisotropy was recorded for 5 μM M39 analog peptide with a substitution equivalent to F1928W in the myosin heavy chain sequence. In the presence of 10 μM S100A4, the anisotropy increased from 0.084 to 0.13. On addition of 10 μM WT M39, the anisotropy decreased towards the free value with an observed rate constant of 0.0014 s^{-1}. Assuming the tryptophan containg peptide has similar binding properties to the native M39, the true dissociation rate constant (equivalent to k_{-2} in scheme 1 of (Badyal et al., 2011)) is ≈ 0.001 s^{-1} as the displacement was only 2/3 complete. Buffer and conditions as in (I). When combined with the association rate constant determined in (J) for the native M39, these data indicate a binding constant ≈ 0.1 nM, comparable with the value from SPR data (H).

(M) Kinetic scheme for the effect of M39 on the binding of Ca^{2+} ions to the two EF2 hand domains of an S100A4 dimer. The scheme is an extension of the single Ca^{2+} binding scheme 1 of (Badyal et al., 2011) and considers that Ca^{2+} binding is non-cooperative but that a single M39 peptide binds to both subunits. Rate constants in black bold are experimental measurements while those in blue are estimates or values required for thermodynamic balance. Note that macroscopic rate constants are used to define the independent Ca^{2+} binding sites to the two sites which give microscopic K_d valuess of 5 μM and 0.5 nM for Ca^{2+} binding to an EF2 hand of the apo and M39-bound S100A4 dimer, respectively. M39 binds to the apo, single Ca^{2+} bound form and double Ca^{2+} bound form of S100A4 with a K_d of 10 mM, 1 μM and 0. 1 nM, respectively.

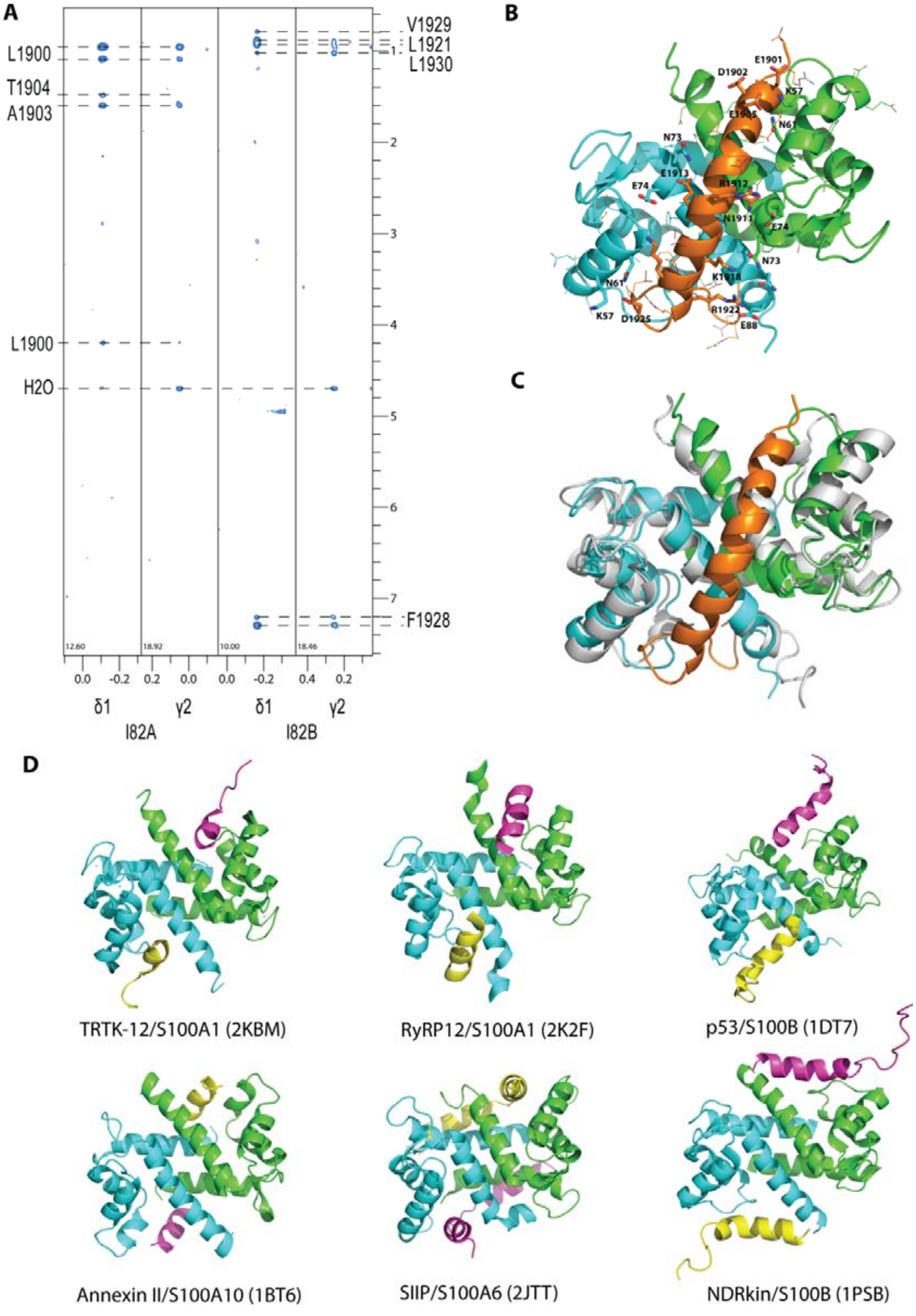
A
L1900
T1904
A1903
L1900
H2O
V1929
L1921
L1930
F1928
δ1
γ2
δ1
γ2
I82A
I82B
B
C
D
TRTK-12/S100A1 (2KBM)
RyRP12/S100A1 (2K2F)
p53/S100B (1DT7)
Annexin II/S100A10 (1BT6)
SIIP/S100A6 (2JTT)
NDRkin/S100B (1PSB)

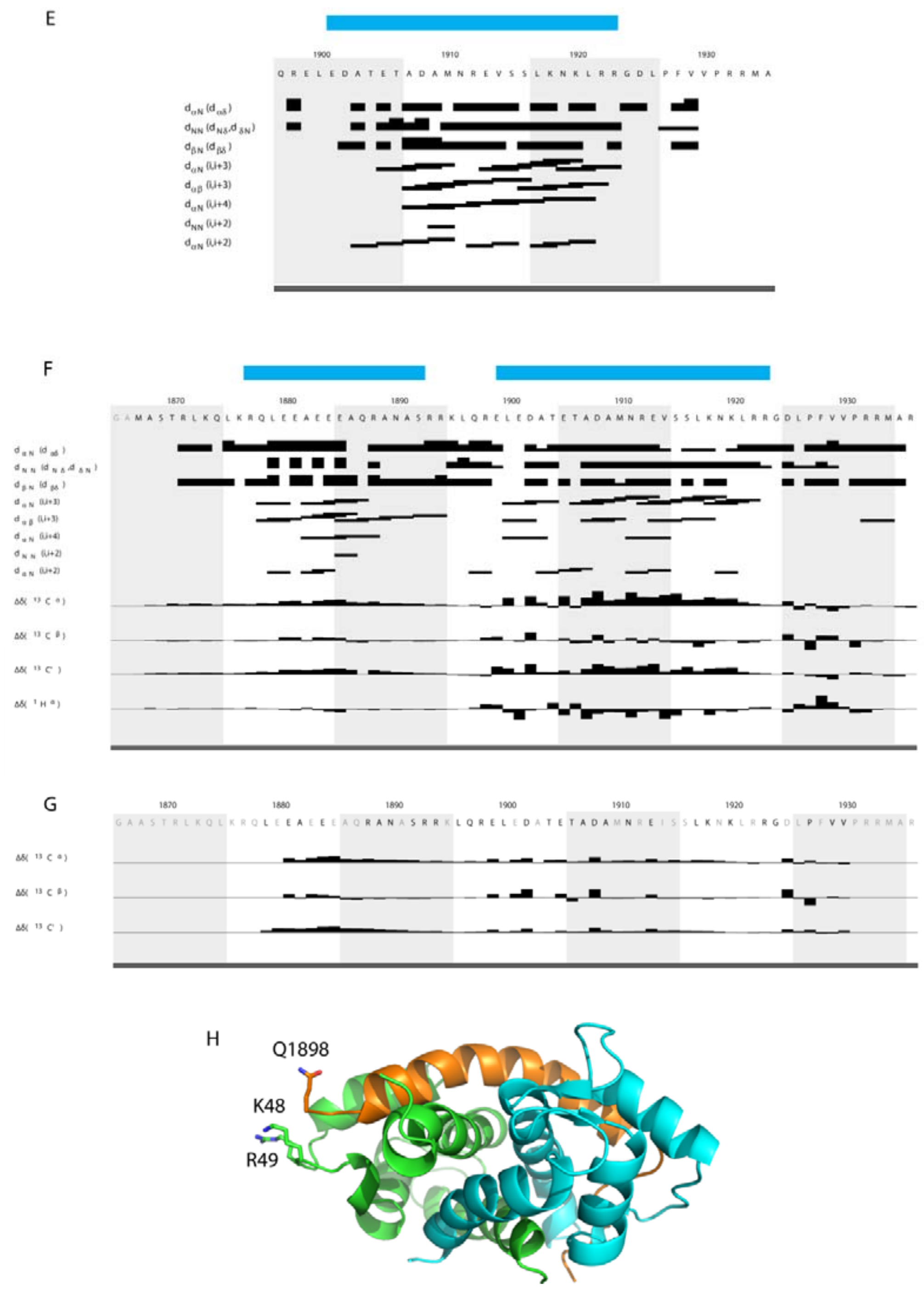

Figure S2, related to Figure 2.

(**A**) Strips from ^{13}C,^{15}N F1-filtered, ^{13}C F_3-edited NOESY-HSQC experiment illustrating the differences in intermolecular NOEs from Me-groups of I82 in the site A (left) and B (right). Assignments of the M39 resonances are marked by dashed lines. See Fig. 2B, D for locations of residues.

(**B**) Superposition of the X-ray structure of the Ca^{2+}-form of S100A4 (PDB ID 3C1V; grey) on the structure of M39/S100A4 complex (S100A4 in green and cyan, M39 in orange).

(**C**) Distribution of charged and hydrophilic residues on the S100A4 surface around the M39 binding site. Residues that are sufficiently close to make contacts are shown as sticks and labelled. S100A4 is coloured in green/cyan, M39 in orange.

(**D**) Comparison of the reported structures of S100 complexes. S100 proteins are shown in green/cyan, ligands in yellow/magenta. The proteins in the complexes and PDB IDs are marked for each structure.

(**E, F**) NMR analysis demonstrates formation of helical structures within myosin peptide in the complexes with S100A4. (**E**) Sequential and medium-range NOE connectivities detected for M39 in 2D ^{12}C-filted NOESY spectrum of u-M39/^{13}C,^{15}N-S100A4. (**F**) Sequential and medium-range NOE connectivities detected for M70 in 3D ^{15}N-edited NOESY-HSQC spectrum of ^{13}C,^{15}N-M70/u-S100A4 complex and backbone ^{13}C chemical shift differences from the random coil values. The positions of helices are marked as blue rectangles above the diagrams.

(**G**) Backbone ^{13}C chemical shift differences from the random coil values for the free M70. Chemical shift values of the free M70 demonstrate that the peptide is unstructured in the S100A4 binding region, but retains a degree of a helical structure in the N-terminal region that does not make contact with S100A4.

(**H**) Proximity of the positively charged side chains of K48 and R49 to the N-terminal end of M39 in the complex.

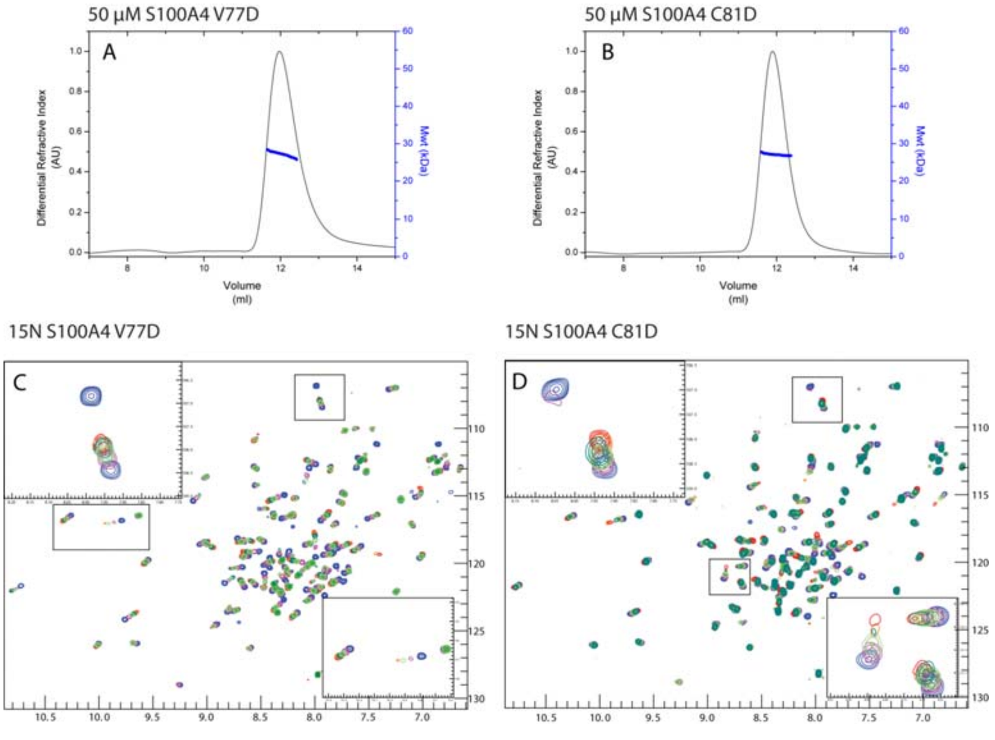

50 µM S100A4 V77D
50 µM S100A4 C81D
A
B
Differential Refractive Index (AU)
Mwt (kDa)
Volume (ml)
15N S100A4 V77D
15N S100A4 C81D
C
D

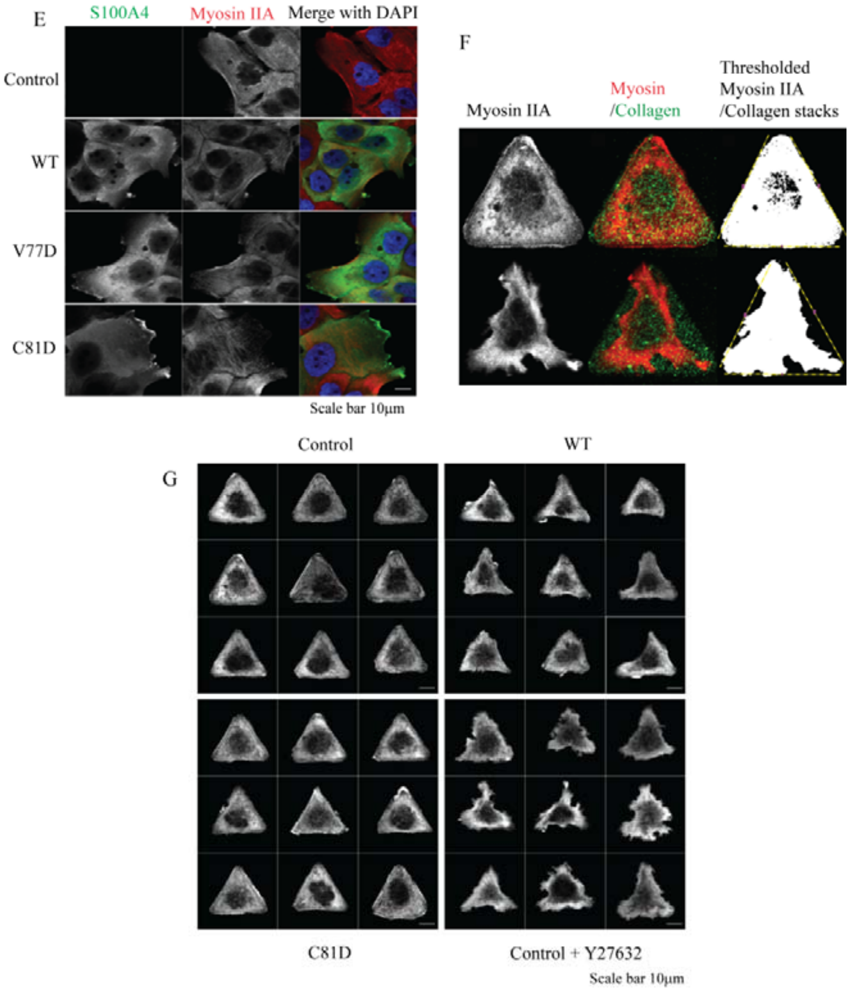

Figure S3, related to Figure 3.

(**A,B**) SEC-MALLS elution profiles and molecular weight distributions for mutant S100A4. The gel filtration elution profiles are similar to that of the WT and the molecular weight measured by MALLS corresponds to the dimeric form (Table S1).

(**C,D**) HSQC spectra of 100 µM ^{15}N-labelled mutants in the presence of different concentrations of u-M39: 0 (blue), 50 (magenta), 100 (light green), 200 (teal), 400 (orange) and 800 µM (red). Many resonances show strong exchange broadening present even at the high M39 excess, as illustrated in the inserts. The complex concentration dependence of broadening and chemical shifts is consistent with the exchange between multiple binding states expected from a disruption of the asymmetrical M39 binding. This prevents accurate evaluation of K_d from the NMR spectra. For many resonances maximum broadening occurs at ~100 µM M39 for V77D and ~200 µM of M39 for C81D. In a two-state exchange model this corresponds to the equimolar concentration of the protein in the free and the bound state, which gives K_d equal to the ligand concentration at the point of maximum broadening, ~100 µM. This estimate agrees with the chemical shift dependence and continuous spectral changes at high ligand excess, as can be seen in the inserts.

(E-G) A431 cells were nucleofected with the pBI empty vector (Control) or pBI vector expressing wild type (WT) or S100A4 mutants, (V77D and C81D). Cells were fixed, permeabilized and stained with antibodies to S100A4 (green) and NMIIA (red). All images were collected with 60x objective on Nikon C1Si confocal laser scanning microscope at the distance closest to the coverslip (scale bar 10µm).

(E). Subcellular localization of NMIIA and WT or mutant S100A4 in nucleofected cells. Images of the nucleofected cells seeded for 24 h on the uncoated glass coverslips and stained with anti-S100A4 (green) or anti-NMIIA antibodies (red) as indicated. Nuclei are visualised by DAPI staining.

(F). An example illustrating analysis of cell shape collapse. For the analysis of the cell shape, nucleofected cells were seeded on the triangle-shaped pattern treated with Y27632 or left untreated and fixed. Samples were double-stained with anti-NMIIA and anti-collagen antibodies to visualise actual cell area and micropattern surface respectively. The images were superimposed and analysed as described in Supplementary Materials and Methods.

(**G**) WT but not the mutant C81D alters the distribution of NMIIA in cells spread on collagen-coated micropatterned glass. Montage of the confocal images of NMIIA-stained cells. 72 h after nucleofection, cells were seeded on the collagen-coated triangle micropatterns. Control cells (Control) were incubated with a ROCK inhibitor Y27632 for 15 min before fixation (Control + Y27632) and stained with the anti-NMIIA antibody.

Table S1. Molecular weights from SEC-MALLS experiments.

Protein[a]	Experimental Mw, kDa	Predicted Mw, kDa
M70	10.6 ±0.04	8.1, monomer
M111	20.1 ±0.16	13.9, monomer
S100A4	27.7 ±0.19	26.2, dimer
M70/A4	34.2 ±0.15[b]	34.3, 1:2 M70:A4
M111/A4	38.3 ±0.11	40.1, 1:2 M70:A4
A4 V77D	27.2 ±0.10	26.2, dimer
A4 C81D	27.1 ±0.08	26.2, dimer

[a] Elution profiles used to estimate molecular weight are shown in Figure.S1.

[b] Corresponds to the main peak. A small shoulder with an average molecular weight of 29.1±1.16 was also detected due to a small excess of free S100A4

[c] Corresponds to the peak 1. An additional peak 2 corresponding to free S100A4 in excess was detected with an average molecular weight of 26.2 ±0.18

Supplementary Methods

Reagents For *in vitro* measurements, proteins were expressed and purified as described previously (Badyal et al., 2011). Spectrophotometric and stopped-flow kinetic methods are also described in this reference, except the fluorescence anisotropy measurements which were carried out in a Fluorolog 3 spectrofluorimeter (Horiba Scientific, Stanmore, U.K.). The M39 peptide and its F1928W variant were purchased from GL Biochem Ltd (Shanghai, China). Molecular weight was confirmed by mass spectroscopy and the purity of >95% by HPLC.

SEC-MALLS Proteins were separated on a Superdex 75 10/300 gel filtration column (GE Healthcare Life sciences) equilibrated in 20 mM 4-morpholineethanesulfonic acid (pH 6.1), 150 mM NaCl, 0.5 mM DTT at 0.7 ml/min. For all proteins 0.5 ml protein solution at a range of concentrations was loaded on the column. Elution was monitored by a Wyatt EOS 18-angle laser photometer (Wyatt Technology), an Optilab rEX refractive index detector and a Jasco UV-2077 Plus UV/Vis spectrophotometer (Jasco). Molar mass determinations were performed using the Astra 5.3.2.16 software (Wyatt Technology).

Isothermal Titration Calorimetry ITC data were collected using an ITC-200 microcalorimeter (GE Healthcare) and analysed by fitting to a single-site binding equation using MicroCal Origin software. The buffer contained 20mM 4-morpholineethanesulfonic acid (pH 6.1), 20 mM NaCl, 5 mM $CaCl_2$, 0.5 mM TCEP. 10 µM of S100A4 in the cell and 75 µM of M39 in the syringe were used in the titrations. The data from three independent experiments were used to evaluate the average values and standard errors for the binding parameters.

Surface Plasmom Resonance (SPR) measurements were conducted on Biacore X100 system using Ni-NTA chip (GE Healthcare). Two His-tags present in the S100A4 dimer resulted in strong coupling with no change in loading after 1h wash. The eluent buffer contained 20mM 4-morpholineethanesulfonic acid (pH 6.1), 150 mM NaCl, 5 mM $CaCl_2$, 0.5 mM TCEP, 50 µM EDTA, 0.005% Surfactant P20. Regeneration buffer contained 350 mM EDTA. 0.5µM S100A4 in the elution buffer was used for coupling. The titration isotherms were analysed on Biacore X100 evaluation software, version 2.0.1. The data from three independent experiments were used to evaluate the average values and standard errors for the binding parameters.

NMR Spectroscopy NMR spectra were measured at 312K using Bruker AVANCE-II 600 and AVANCE-II 800 MHz spectrometers both equipped with CryoProbes. Proton chemical shifts were referenced to external DSS. The ^{15}N and ^{13}C chemical shifts were referenced indirectly using recommended gyromagnetic ratios (Wishart et al., 1995). Spectra were processed with TopSpin (Bruker) and analysed using ANALYSIS (Vranken et al., 2005). 3D HNCO, HN(CA)CO, HNCA, HN(CO)CA, HNCACB and HN(CO)CACB experiments were used for the sequential assignment of the backbone NH, N, CO, C^{α} and C^{β} resonances. Side chain assignments were obtained using 3D HBHA(CO)NH, HBHANH, H(C)CH-TOCSY and (H)CCH-

TOCSY experiments. Aromatic side-chains were assigned using ^{13}C-resolved 3D NOESY-HSQC. The resonances of u-M39 were assigned using ^{13}C,^{15}N-filtered 2D TOCSY and NOESY experiments.

NMR Structure Calculations Distance restraints were derived from the following experiments: 3D ^{15}N-edited NOESY-HSQC (800 MHz, 100ms), ^{13}C-edited NOESY-HSQC (800MHz, 100ms) optimised for detection of aliphatic resonances and ^{13}C-edited NOESY-HSQC (800MHz, 100ms) for aromatic resonances, 3D ^{13}C,^{15}N F1-filtered, ^{13}C F_3-edited NOESY-HSQC and 2D ^{13}C,^{15}N F1-filtered NOESY. 3D spectra were collected for u-M39/^{13}C,^{15}N-S100A4, u-M70/^{13}C,^{15}N-S100A4 and ^{13}C,^{15}N -M70/u-S100A4, 2D experiments recorded for u-M39/^{13}C,^{15}N-S100A4. All NOESY peaks were picked semi-automatically in ANALYSIS with noise and artefact peaks removed manually. Cross-peak intensities were used to evaluate the target distances. Dihedral restraints (Φ/Ψ) were generated using DANGLE module of ANALYSIS software.

The structure of the complex was calculated with Aria 1.2 (Linge et al., 2001) in two stages. In the first stage intra-molecular NOEs for S100A4 were assigned on the basis of chemical shifts separately for each spectrum and used as uncalibrated restraints. The ambiguity of these restraints was resolved in the Aria cycles with the violation tolerances set to 5.0, 2.0, 1.0, 0.5, 2.0, 0.5, 0.5 Å for iterations 2-8, respectively, with iteration 1 containing the initial models. Intra-molecular NOEs for M39 were assigned on the basis of chemical shifts and calibrated to the average distance of 3.2 Å. Inter-molecular NOEs observed in the filtered experiments were assigned on the basis of chemical shifts and cross-validated in all the spectra collected for u-M39/^{13}C,^{15}N-S100A4, u-M70/^{13}C,^{15}N-S100A4 and ^{13}C,^{15}N -M70/u-S100A4. Only the NOEs with consistent assignments in all the spectra were converted into distance restraints and calibrated to the average distance of 3.2 Å. Assignments and calibration for both intra- and inter-molecular NOEs of M39 were fixed in the Aria cycles at this stage. Unassigned intra-molecular NOEs for S100A4 were checked at the end of the Aria cycle, chemical shifts and resonance assignments were adjusted where necessary and calculation repeated using the lowest energy structure from the previous. At this and subsequent stage dihedral angle restraints and symmetry restraints for N-terminal half of S100A4 (residues 1-44) were applied.

At the second stage intra-molecular restraints for S100A4 from the first stage were used as fixed restraints. Inter- and intra-molecular NOEs for M39 were assigned independently in each spectrum and used as un-calibrated restraints. The ambiguity of these restraints was resolved in the Aria cycles with the violation tolerances as in the first stage. The lowest energy structures of the complex generated in the first stage were used as starting models. Unassigned NOEs for M39 were checked at the end of the Aria cycle, chemical shifts and resonance assignments were adjusted where necessary and calculation repeated using the lowest energy structure from the previous cycle to avoid bias to the initial model. At the intermediate stages 30 structures were calculated at each Aria iteration with 10 structures used to resolve ambiguities of the assignments. Finally 200 structures were calculated, 30 best refined in the in the presence of explicit water molecules, 20 lowest structure used for the analysis and PDB deposition. Statistics of the structure calculation are presented in Table 1.

Model of NMIIA coiled-coil structure. The coiled-coil structure of the C-terminal NMIIA fragment was modelled using the backbone geometry of the crystal structure of myosin V coiled-coil (PDB ID 2DFS). Side-chains were added and the structure minimised using CNS software (Brunger et al., 1998). The model of NMIIA coiled-coil in complex with S100A4 was generated using Aria protocols from a combination of distance restraints calculated for the NMIIA coiled-coil region and M39/S100A4 complex. No restraints were used for the transition region S1893-L1897.

Plasmids Wild type or mutant S100A4 cDNA were cloned into a DOX (doxycycline)-inducible pBI vector (BD Bioscience, USA). All mutants were generated by using QuikChange Site-Directed Mutagenesis kit (Agilent Ltd., UK) with the following primers

5’ ACTTCCAAGAGTACTGTGACTTCCTGTCCTGCATCG 3’ and

5’ CGATGCAGGACAGGAAGTCACAGTACTCTTGGAAGT 3’- for V77;

5’ GAGTACTGTGTCTTCCTGTCCTGCATCGCCATGATG 3’ and

5’ CATCATGGCGATGCAGGACAGGAAGACACAGTACTC 3’ -for C81D.

All constructs were validated by sequencing.

Cell culture and transfection A clone of human epidermoid carcinoma A431 cells with the stable expression of the TET-responsive transcriptional activator, rtTA, was used throughout the study (Andersen et al., 2005). Cells were maintained in DMEM supplemented with 10% foetal bovine serum (FBS), 1% penicillin/streptomycin at 37°C in a humidified incubator under an atmosphere of 5% CO_2. All reagents were purchased from PAA, UK. For transfection, 2.5×10^6 cells were mixed with 2µg of plasmid DNA in 100µl of buffer V (Lonza, Switzerland) and nucleofected (T020 program) using Amaxa Nucleofector (Lonza, Switzerland) according to the manufacturer’s protocol. After transfection, cells were grown in the presence of 2 µg/ml DOX (Sigma-Aldrich, USA).

Cell migration assay 48 h after nucleofection cells were harvested, and 5×10^4cells were resuspended in 200µl of serum-free medium and seeded onto fibronectin-coated (5µg/ml of fibronectin, Sigma-Aldrich, UK) inserts (8.0µm inserts from BD Biosciences, UK). 10% serum was used as a hemoattractant. The cells were allowed to migrate for 24 h and visualised with Hema Gurr staining kit (VWR International, UK). Non-migrated cells were removed by scrapping the upper side of the membrane with a cotton swab. Cells were counted with a 20x/0.45 Plan Fluor objective on Nikon Eclipse TE 2000-U microscope. Migration rate was expressed by normalization of the average number of cells in 10 fields to the number of cells in a control. Samples were run in triplicate, and three independent experiments were performed.

Micropattern printing The equilateral triangular shape patterns were prepared with a solution of 300 μg/ml of rat collagen type I (BD Biosciences, USA) in PBS. The non-adhesive areas outside the pattern were backfilled with polyethylene glycol, PLL-g-PEG (SUSOS, Switzerland) for 1 h.

Image Analysis Image analysis (comparative length of the of the peripheral stress fibers, relative area and cell shape analysis) was carried out using the ImageJ Version 1.44 software (NIH, Bethesda, USA). A custom macro was used to analyse cell shape in which the edge collapse was defined as an edge that deviates by more than 50% from the micropattern boundary. Essentially, three lines of 41 μm, corresponding to each edge of the micropattern boundary were superimposed over thresholded, 8-bit binary images of stacks of cells. The modal pixel value was calculated along the length of each line such that a value of 0 (no cell) or 255 (a cell) was recorded for each edge. If 50% or more pixels contained no cell then an edge was considered collapsed. The number of collapsed edges was divided by the total number of edges for each population and expressed as a percentage. The micropattern boundary was superimposed (yellow line) over the binary image and the modal pixel value (0 – no cell; 255 – a cell) was calculated along this length.

Statistical Analysis Statistical significance of all *in vivo* assays was assessed with one-way ANOVA followed by a Dunnett's Multiple Comparison test using Prism 5 software.

References

Andersen, H., Mejlvang J., Mahmood S., Gromova I., Gromov P., Lukanidin E., Kriajevska M., Mellon J.K., and Tulchinsky E. (2005).Immediate and delayed effects of E-cadherin inhibition on gene regulation and cell motility in human epidermoid carcinoma cells. Mol Cell Biol *25*, 9138-9150.

Badyal, S.K., Basran J., Bhanji N., Kim J.H., Chavda A.P., Jung H.S., Craig R., Elliott P.R., Irvine A.F., Barsukov I.L. et al. (2011).Mechanism of the Ca(2)+-dependent interaction between S100A4 and tail fragments of nonmuscle myosin heavy chain IIA. J Mol Biol *405*, 1004-1026.

Brunger, A.T., Adams P.D., Clore G.M., DeLano W.L., Gros P., Grosse-Kunstleve R.W., Jiang J.S., Kuszewski J., Nilges M., Pannu N.S. et al. (1998).Crystallography & NMR system: A new software suite for macromolecular structure determination. Acta Cryst D *54*, 905-921.

Linge, J.P., O'Donoghue S.I., and Nilges M. (2001).Automated assignment of ambiguous nuclear overhauser effects with ARIA. Methods Enzymology *339*, 71-90.

Vranken, W.F., Boucher W., Stevens T.J., Fogh R.H., Pajon A., Llinas P., Ulrich E.L., Markley J.L., Ionides J., and Laue E.D. (2005).The CCPN data model for NMR spectroscopy: Development of a software pipeline. Proteins *59*, 687-696.

Wishart, D.S., Bigam C.G., Yao J., Abildgaard F., Dyson H.J., Oldfield E., Markley J.L., and Sykes B.D. (1995).H-1, C-13 AND N-15 Chemical shift referencing in biomolecular NMR. J of Biomol NMR *6*, 135-140.
